# Non-mantle-plume process caused the initial spreading of the South China Sea

**DOI:** 10.1038/s41598-020-65174-y

**Published:** 2020-05-22

**Authors:** Xun Yu, Zhifei Liu

**Affiliations:** State Key Laboratory of Marine Geology, Tongji University, Shanghai, 200092 China

**Keywords:** Ocean sciences, Solid Earth sciences

## Abstract

The mantle plume process is thought to be the prevailing dynamic mechanism for the South China Sea opening, but controversy persists due to the lack of critical evidence of magma in the initial seafloor spreading. International Ocean Discovery Program (IODP) Expedition 367 successfully recovered at Site U1500 the mid-ocean ridge basalt (MORB) representing the magma activity of the initial spreading of the South China Sea during the earliest Oligocene. Here we present the whole-rock and olivine phenocryst geochemistry of the basalts to constrain the potential influence of the Hainan mantle plume on the evolution of the South China Sea. Major and trace elemental compositions indicate that the basalts were mainly influenced by fractional crystallization of olivine and formed by melting of a spinel peridotite source without any pyroxenite in mantle source. The calculated mantle potential temperature of those most primitive basalts is much lower than plume-related MORB of Iceland, but similar to normal MORB elsewhere. Both lithological composition and mantle potential temperature clearly contradict with the mantle plume model, signifying that the mantle plume didn’t exist at the earliest Oligocene. Therefore, the initial spreading of the South China Sea should be caused by non-plume processes, most likely by the westward subduction of the Pacific Plate.

## Introduction

Dynamic processes of continental breakup and subsequent initial generation of igneous oceanic crust have been identified as two end-members: magma-rich type and magma-poor type^1,2^. For the magma-rich type, the rifted margin is characterized by massive igneous activity in a relatively short period of time (~1–3 Myr) during breakup and initial seafloor spreading, which is caused by mantle plume. The pair of conjugate margins of Greenland and northwest Europe is a typical example^3^. For the magma-poor type, weakening of mantle lithosphere and facilitating of plate rupture is caused by serpentinization over a period of time which is a result of tectonic extension and mantle exhumation. The Newfoundland and Iberia conjugate margin is a typical example^4^. However, the South China Sea (SCS) margin doesn’t meet the expected characteristics of neither magma-rich nor magma-poor types, instead showing a rapid transformation from continental breakup to initial spreading without massive magmatism^5^. Revealing the formation process of such a unique type margin can help us understand the dynamic mechanism of the marginal sea evolution. Meanwhile, it also provides an important supplementary knowledge for the continental breakup and oceanic formation on the Earth.

Various models have been put forward to explain the formation of the SCS, such as tectonic extrusion by India-Eurasia collision^6,7^, extension related to mantle plume upwelling^8–10^, and regional extension by subduction and retreat of the Pacific plate^11^. Among them, the mantle plume is considered as the most popular model^10,12–14^. The evidence include: (1) seismic observations indicate a low-velocity zone beneath the SCS that extends to the lower mantle, supporting the existence of a deep plume^15,16^; (2) the mantle potential temperatures of Hainan ocean island basalts (OIB), as well as the olivine crystallization temperatures of mid-ocean ridge basalts (MORB) from the east sub-basin of the SCS, are similar to those of hotspot-related OIB and higher than those of normal MORB (N-MORB)^12,17^; (3) The EM2-like Sr, Nd, and Pb isotopic geochemistry of SCS OIB and MORB are consistent with the isotopic features of Hainan OIB^13,14^, which are considered to origin from a deep mantle plume^18,19^.

However, the MORB samples reported from Site U1431 (IODP Expedition 349) represent oceanic crust that formed toward the end of extension (16–15 Ma) of the SCS basin^10,13,14,20^. Intraplate basalts like Hainan OIB are much younger than the spreading cessation age of the SCS basin^8^. As a result, the thermal state and geochemical nature of mantle sources which were recovered from above samples cannot decide the role of Hainan plume in the early evolution history of the SCS basin. In addition, the recent drilling results suggest that the SCS basin experienced a rapid transformation from continental breakup to igneous oceanic crust without abundant magmatism^5^. The above feature seems inconsistent with plume-induced opening model as characterized by excessive magmatism like magma-rich type margin in the North Atlantic^5^. Therefore, to determine whether the Hainan plume played an important role in opening of the SCS basin, obtaining the mantle potential temperature (*T*p) of the upper mantle and understanding the mantle source lithology of basalts formed at the initial spreading stage is the key. In this study, major- and trace-element whole-rock and olivine phenocryst data are reported for the basalts of IODP Expedition 367 Site U1500. These basalts represent oceanic crust produced during the initial spreading of the SCS at ~33 Ma^5,21^. The geochemical data of Site U1500 basalts are used to determine the mantle source lithology and *T*p of the upper mantle that produced the basalts, as well as the role of the Hainan plume on the formation and evolution of the SCS basin.

**Figure 1:**
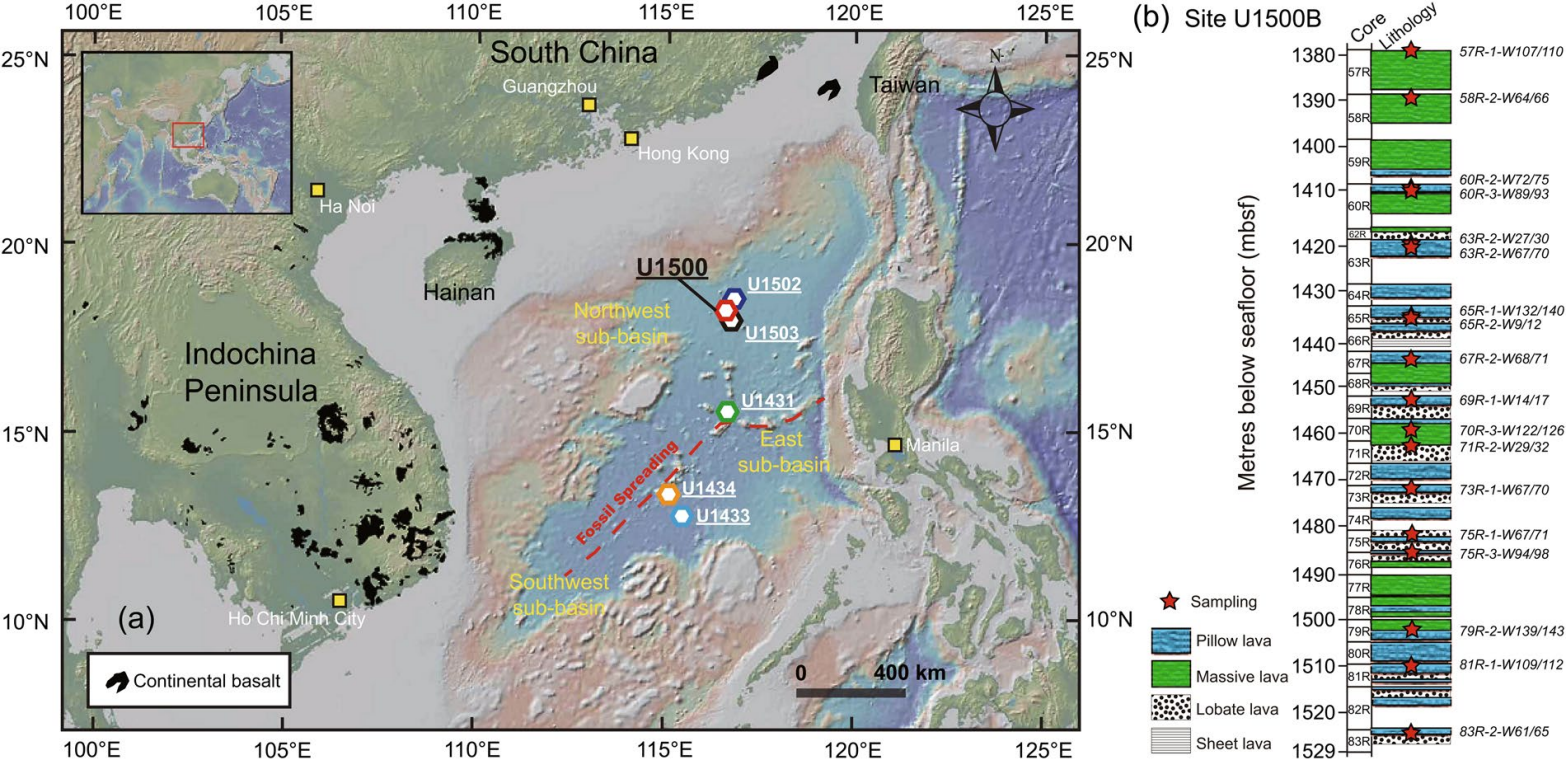
Topographic map of the South China Sea (SCS) and sampling location (**a**), and stratigraphy of recovered core (**b**). Locations of sites which drilled into basement rocks are shown. Sites U1431, U1433, and U1434 are from IODP Expedition 349^22^. Site U1500 is from IODP Expedition 367, Site U1502 is from IODP Expedition 368, and Site U1503 is from IODP Expedition 368X^21,23^. Distribution of Cenozoic intraplate volcanisms is also shown^8^. Figure (a) made with GeoMapApp 3.6.6 (www.geomapapp.org).

The SCS is located at the junction of the Eurasian, Pacific, and Indo-Australian plates^5,21–23^ (Fig. 1a). The deep-water basin of the SCS can be divided into the east sub-basin, the southwest sub-basin, and the northwest sub-basin (Fig. 1a). Among them, the east sub-basin and southwest sub-basin are two main deep basins, which are separated by the N-S trending Zhongnan Fault^22^. The east sub-basin was formed by north-south spreading between 33–15.5 Ma and the southwest sub-basin was formed by northwest-southeast spreading between 24–16 Ma^24^. After the spreading cessation, intraplate volcanisms formed within and around the SCS^8,25–27^ (Fig. 1a). Four sites were drilled by IODP Expeditions 367 and 368 in the northern margin of the east sub-basin, one on the outer margin high and three seaward on the outer margin high basement ridges^21^. These ridges are within the continent-ocean transition zone going from outer margin high to the steady-state oceanic crust of the SCS. The seafloor in this region is thought to have formed at ~32–30 Ma, the half-spreading rate of which was ~3.6 cm/y^24^. Site U1500 is the most seaward site (Fig. 1a), which can stand for the early stage of magmatism during seafloor spreading^21^. 149.9 m of igneous rocks were cored below sedimentary section and a total of 114.92 m of basalt was recovered^21^ (Fig. 1b). The basalts are divided into two igneous subunits according to flow boundaries to distinguish an upper massive lava flow sequence (27.28 m thick) from a lower, predominantly pillow lava flow succession (122.62 m thick) with subordinate thin (<6 m) interbedded lobate, sheet, and massive lava flows. The pillow lobes are well preserved and are separated by chilled, glassy margins and also claystone^28^. Basalt samples remain similar in texture and mineralogical composition, varying from nonvesicular to moderately vesicular, cryptocrystalline to fine grained, and aphyric to highly (olivine-) plagioclase phyric with a hypocrystalline groundmass^28^. Plagioclase phenocrysts are found throughout these basalts with olivine being an occasional phenocryst, and rare clinopyroxene phenocrysts are identified (Supplementary Fig. S1a). The abundance of plagioclase and olivine phenocrysts increases downhole, reaching a peak of 20% to 35% for plagioclase and 5% to 10% for olivine^28^. In this study, 18 basalt samples were collected from Site U1500 and they are fresh in hand specimen and thin sections (Supplementary Fig. S1b).

## Geochemical results

**Figure 2:**
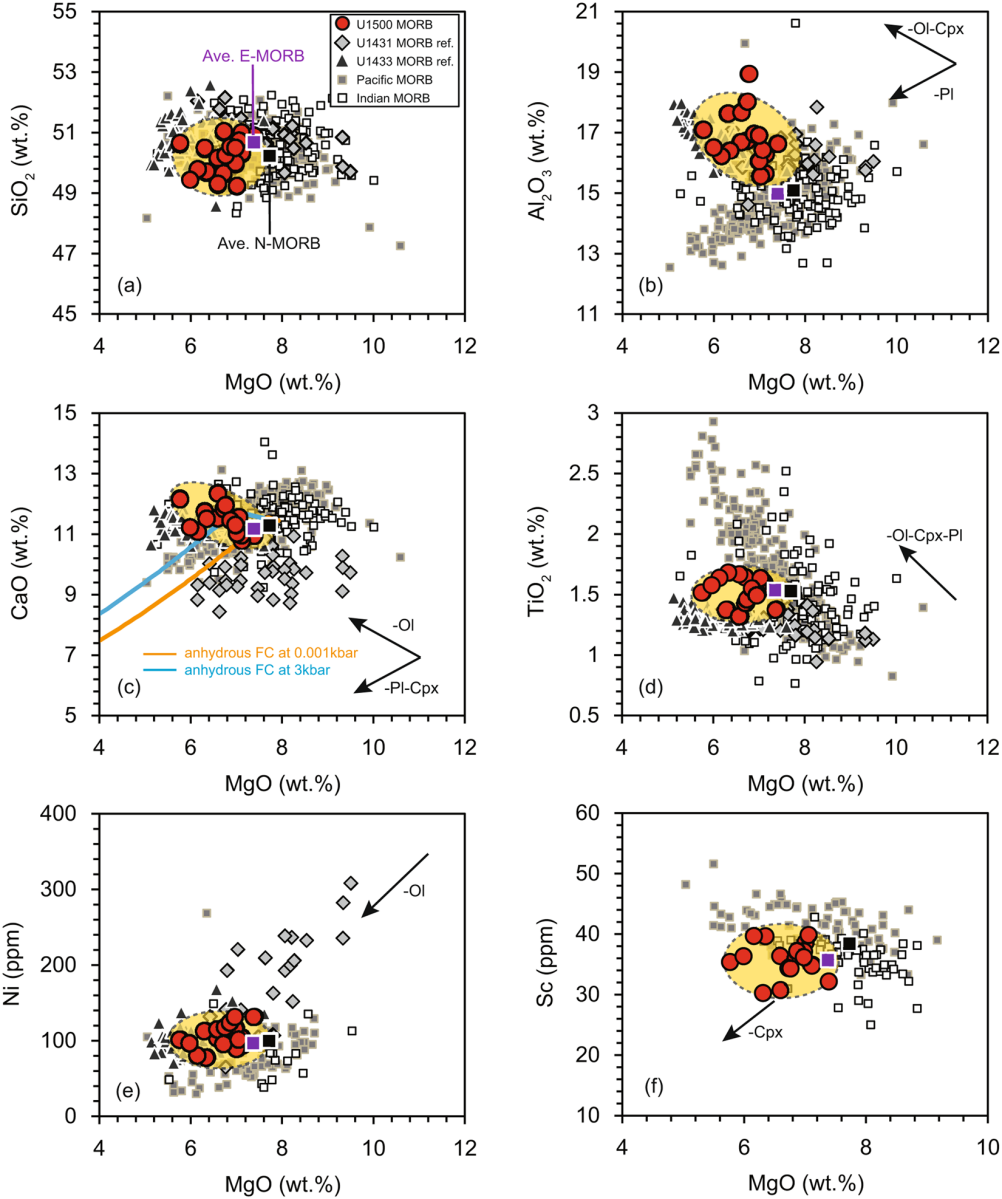
Plots of SiO_2_ (**a**), Al_2_O_3_ (**b**), CaO (**c**), TiO_2_ (**d**), Ni (**e**), and Sc (**f**) versus MgO for Site U1500 basalt samples. Fractional crystallization simulation by “Petrolog 3” software^59^ is present in plot (c) as a function of olivine, plagioclase, and clinopyroxene removal for Site U1500 basalts at pressures of 0.001 kbar and 3 kbar. Average N-MORB is used as the starting magma during the modeling. Reference data for basalt samples from Sites U1431 and U1433 are referred to references^10,14^. Average elemental data for N-MORB and E-MORB are from Gale *et al*.^60^. Data for global MORB (Pacific Ocean MORB and Indian Ocean MORB) are derived from Petrological Database (http://www.earthchem.org/petdb).

Site U1500 basalts are classified as tholeiitic basalts on an Na_2_O + K_2_O versus SiO_2_ diagram, consistent with MORB elsewhere and with basalts from Sites U1431 and U1433 in the eastern and southwestern sub-basins of the SCS, respectively (Supplementary Fig. S2a). Site U1500 basalts are classified as normal MORB (N-MORB) and transitional MORB (T-MORB) on the MORB classification diagram, whereas Site U1431 basalts are identified as N-MORB (Supplementary Fig. S2b). Site U1500 basalts have higher Al_2_O_3_ and CaO contents than those of average N-MORB with similar SiO_2_ and TiO_2_ contents (Fig. 2a–d). Ni and Sc contents of Site U1500 basalts are similar to those of average N-MORB (Fig. 2e,f). In comparison, Site U1431 basalts have lower CaO, TiO_2_, and higher Ni contents compared with average N-MORB and Site U1500 basalts (Fig. 2c–e). The primitive-mantle-normalized trace-element patterns of Site U1500 basalts and average N-MORB are similar (Supplementary Fig. S3a) but differ from those of basalts from Sites U1431 and U1433 (Supplementary Fig. S3b). Furthermore, basalts from Sites U1431 and U1433 are characterized by positive Sr and Eu anomalies, but Site U1500 basalts lack of Sr and Eu anomalies (Supplementary Fig. S3). Nb content is correlated with the contents of the rare-earth elements (REEs; e.g., La and Yb) (Supplementary Fig. S4a) and of the other high-field strength elements (HFSEs; e.g., Hf) (Supplementary Fig. S4b, c). In contrast, Nb is not correlated with some of the large-ion-lithophile elements (LILEs; e.g., Ba and U) (Supplementary Fig. S4d). In summary, Site U1500 basalts can be classified as MORB and represent oceanic crust formed during initial spreading of the SCS basin. Bulk major- and trace-element data are provided in Supplementary Table S1, and the details of methods are provided in the Supplementary Materials.

CaO contents of most of the olivines are 0.22–0.40 wt.%, higher than those of olivines from mantle xenoliths (CaO <0.1 wt.%)^29,30^. Ni abundance decreases with decreasing forsterite (Fo) content, as expected for olivine fractional crystallization (Supplementary Fig. S5a). The Fo content of olivine was plotted as a function of the Mg# (100*molar Mg/(Mg + Fe^2+^)) of the host basalt (Supplementary Fig. S5b), assuming that the Fe^3+^ / total Fe ratio of the basalt is 0.1. The Fe–Mg exchange partition coefficients of olivine and basaltic melt (K_D_ = (Fe/Mg)°^l^/(Fe/Mg)^liq^) are well constrained by experiments to 0.30 at 1 atm and 0.31–0.34 at 5–15 kbar^31^. The olivine of our sample plots within the equilibrium field (Supplementary Fig. S5b). The major-element composition of olivine was analyzed by electron probe microanalysis (Supplementary Table S2), and the details of method are described in the Supplementary Materials.

## Discussion

### Effects of seawater alteration

Seawater alteration commonly affects the composition of MORB^32,33^, and its effects must be evaluated prior to interpretation of the geochemical data. The petrographic observations and loss on ignition (LOI) values (<3 wt.%) indicate that seawater alteration was not extensive (Supplementary Fig. S1; Table S1). Furthermore, K_2_O/Nb, which is commonly used as a proxy for seawater alteration^34^, is not correlated with LOI, consistent with minimal seawater alteration, except for sample 57R-1-W107/110 and 75R-3-W94/98 (Supplementary Fig. S6a). Some samples (e.g., 60R-2-W72/75, 65R-1-W132/140, and 79R-2-W139/143) have variable LILEs (e.g., U) on plots of LOI versus element content (Supplementary Fig. S6b), indicating that seawater alteration affected these LILEs. However, the LOI values are not correlated with Ni, Ba, the REEs, or the HFSEs (Supplementary Fig. S6c–f), meaning that most elements were not affected by seawater alteration. Finally, the correlations amongst Nb and a range of elements also indicate that seawater alteration was not extensive (Supplementary Fig. S4). Elements affected by alteration are not well correlated with Nb because seawater alteration affects the concentrations of fluid-mobile elements more than it affects those of fluid-immobile elements^34^. The positive correlations among the fluid-immobile incompatible elements (e.g., Nb) and the fluid-mobile incompatible elements (e.g., La) and the HFSEs (e.g., Hf and Th) indicate that seawater alteration did not affect the whole-rock composition significantly, except for samples 60R-2-W72/75 and 65R-1-W132/140 (Supplementary Fig. S4d). Therefore, we can conclude that the effect of seawater alteration was negligible for most of the samples.

### Shallow magmatic processes

#### Effects of fractional crystallization

Basalts of Site U1500 are more enriched in the strongly incompatible elements (e.g., Ba and Nb) compared with basalts from Site U1431 and average N-MORB (Supplementary Fig. S3), which might be a consequence of fractional crystallization. Olivine fractionation increases the contents of the strongly incompatible elements, SiO_2_ and Al_2_O_3_, and decreases MgO content (Fig. 2b). Simultaneous fractionation of olivine and clinopyroxene increases the abundance of the strongly incompatible elements (e.g., Ba and Nb) and decreases the contents of MgO, CaO, and Ni (Fig. 2c,e). Simultaneous fractionation of olivine and plagioclase increases the contents of the strongly incompatible elements and decreases Al_2_O_3_ content (Fig. 2b). Site U1500 basalts are characterized by decreasing in MgO and Ni contents (Fig. 2e) with increasing Al_2_O_3_ and CaO contents (Fig. 2b,c). The whole-rock CaO/Al_2_O_3_ ratios and Sc abundance (Fig. 2f) remain nearly constant, and the basalts show slight negative correlation between MgO and CaO (Fig. 2c), suggesting that clinopyroxene fractionation was limited. In addition, the negative correlation between MgO and Al_2_O_3_ (Fig. 2b), and the lack of negative Eu and Sr anomalies (Supplementary Fig. S3) suggest that plagioclase fractionation did not take place. Fractional crystallization simulation has been implemented to model the MgO and CaO compositional variation as a function of olivine, plagioclase, and clinopyroxene removal for Site U1500 basalts at different pressures (0.001 kabr versus 3 kbar; Fig. 2c). Because Ca is compatible in clinopyroxene, fractional crystallization of clinopyroxene could account for the decreasing CaO content. The difference in pressures (0.001 kbar versus 3 kbar) can determine the break point without changing the fate of CaO (Fig. 2c). Therefore, fractional crystallization of olivine occurred without fractionation of plagioclase or clinopyroxene. The strongly incompatible elements (e.g., HFSEs and REEs) are incompatible in olivine, whereas Mg and Ni are compatible, so the main effect of olivine fractional crystallization is to decrease the contents of Mg and Ni and increase the contents of the strongly incompatible elements. The basalt whole-rock compositions (incompatible trace element ratios) are suitable to infer compositional characteristics of their mantle source and those acquired by melt-rock interaction.

#### Effects of melt–rock interaction

**Figure 3:**
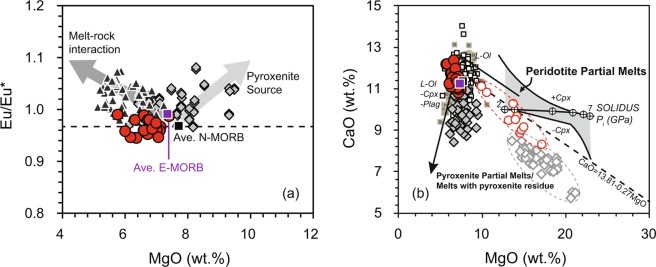
Eu/Eu* (**a**) and CaO (**b**) versus MgO for Site U1500 basalt samples. In (**a**), elemental anomalies are calculated as follows: Eu/Eu* = Eu_N_/(Sm_N_ × Gd_N_)^0.5^, N here stands for chondrite normalized. In (**b**), the gray dotted line separating pyroxenite melts from peridotite melts is from Herzberg and Asimow^53^. Lavas with CaO contents lower than those defined by the dotted line are potential pyroxenite-bearing partial melts. Reference data for basalt samples from Sites U1431 and U1433 are referred to references^10,14^. In (**b**), the calculated primitive melt compositions for Site U1500 basalts are shown in red circles while calculated primitive melt compositions for Site U1431 are shown in grey diamonds. Average elemental data for N-MORB and E-MORB are from Gale *et al*.^60^. Data for global MORB (Pacific Ocean MORB and Indian Ocean MORB) are derived from Petrological Database (http://www.earthchem.org/petdb). Symbols are the same as in Fig. 2.

Melt–rock interaction in the oceanic lithosphere commonly affects the major- and trace-element compositions of MORB formed at slow-spreading ridges prior to eruption^35^. Interaction between MORB melts and oceanic lithospheric mantle increases the MgO and Al_2_O_3_ contents of MORB, decreases its SiO_2_ content, and increases the contents of the strongly incompatible elements^36,37^. Melt–rock interaction in the lower oceanic crust can produce MORB with high Al_2_O_3_ and CaO contents, depleted strongly incompatible elements, and positive Sr and Eu anomalies^38^. Site U1500 basalts are characterized by higher Al_2_O_3_ and CaO contents than average N-MORB. However, Site U1500 basalts are also featured by lower MgO contents than average N-MORB with similar SiO_2_ and TiO_2_ contents (Fig. 2a,d), and without any enrichment of strongly incompatible elements (Supplementary Fig. S3). The above geochemical variations cannot be interpreted by process of melt-rock interaction in oceanic lithospheric mantle. In addition, Site U1500 basalts do not have positive Sr and Eu anomalies, in contrast to the elemental anomalies as shown by Site U1433 basalts, which were affected by melt–rock interaction in the lower oceanic crust^39^ (Supplementary Fig. S3a). Furthermore, melt-rock interaction influenced Site U1433 basalts are characterized by negative correlation between MgO and Eu/Eu*; however, the MgO content of basalts of Site U1500 is not correlated with Eu/Eu* (Fig. 3a), excluding a role for gabbro in their formation. The above observations together indicate that melt–rock interaction did not affect the geochemical compositions of the basalts of Site U1500.

### Peridotite mantle source for Site U1500 basalts

The geochemistry of basalt can reflect the nature of its mantle source if the effects of shallow processes (e.g., seawater alteration, fractional crystallization, and melt–rock interaction) can be recognized or excluded. Here, a comparison of our new data with high-temperature experimental data and whole-rock and olivine geochemical data is used to infer that the mantle source lithology of Site U1500 basalts is spinel peridotite.

#### High-temperature experimental data

The contribution of mafic eclogite and pyroxenite to the source of basalts can be constrained by using the major-element compositions of the basalts and high-temperature experimental data^40^. Calcium is highly incompatible within olivine (D_Ca_^Ol^ = 0.02)^41^, but compatible within clinopyroxene (D_Ca_^Cpx^ = 1.82–1.95)^42^. Therefore, the CaO content of a melt produced by low-degree partial melting of a pyroxenite source is lower than that of a melt derived from peridotite^43^. Primary melts of peridotite produced at pressures up to 7 GPa have high CaO contents (~10 wt.%), irrespective of the fertility of the source peridotite^44^. Site U1500 basalts and the calculated primitive melt compositions of Site U1500 basalts are plotted within the field of peridotite melt on a plot of CaO versus MgO (Fig. 3b). They have similar geochemical features to most of the Pacific and Indian Ocean MORB and average N-MORB (Fig. 3b). In comparison, at a given MgO content, Site U1431 basalts, which have been identified as the melting result of pyroxenite source^10^, have CaO contents lower than average N-MORB and are plotted within the field of pyroxenite melt. Therefore, the comparison with high-temperature experimental data indicate that Site U1500 basalts had a peridotite, rather than a clinopyroxene-rich, source.

#### Whole-rock and olivine geochemistry

**Figure 4:**
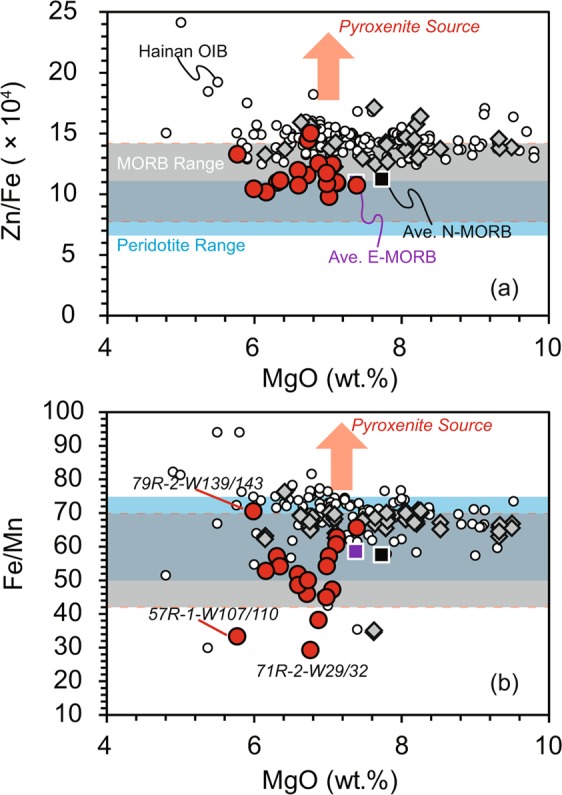
Relationship between (**a**) Zn/Fe (×10^4^), (**b**) Fe/Mn and MgO contents for Site U1500 basalt samples. Data of Site U1431 MORB are referred to Zhang *et al*.^10,14^, and data of Hainan OIB^17,61,62^ are collected for comparison which are represented by white circles. Average elemental data for N-MORB and E-MORB are from Gale *et al*.^60^. Fields for peridotite and global MORB are from Le Roux *et al*.^45^. Symbols are the same as in Fig. 2.

Le Roux *et al*.^45,46^ showed that Zn/Fe and Fe/Mn do not fractionate among olivine, orthopyroxene, and melt, but fractionate strongly if garnet or clinopyroxene are involved in melting or crystallization. Davis *et al*.^47^ used experiments to constrain the partition coefficients of Zn, Fe, and Mn in partial melts of peridotite. Those authors demonstrated that values of (Zn/Fe) × 10^4^ > 13 and Fe/Mn > 62 are characteristic of a non-peridotite source for natural OIBs, and that partial melts of eclogite or garnet pyroxenite have higher Zn/Fe and Fe/Mn values compared with partial melts of peridotite. The Hainan basalts are thought to be formed by melting of a pyroxenite source^17^. The values of (Zn/Fe) × 10^4^ and Fe/Mn of Hainan basalts with MgO > 7.5 wt.% are 12.4–17.1 and 57.4–76.7, respectively, higher than the values for MORB and typical peridotite data (Fig. 4). In contrast, Site U1500 basalts are characterized by similar and lower Zn/Fe and Fe/Mn values than those of average N-MORB and are plotted within the fields of MORB and peridotites^45^ (Fig. 4), consistent with a peridotite source for these basalts.

We used the composition of olivine to provide additional constraints on the source of Site U1500 basalts. Olivine is the first silicate mineral to crystallize from almost all mantle-derived magmas as they ascend towards the surface. The presence of pyroxenite or eclogite within the mantle source is recorded by olivine phenocrysts with high Ni contents^48^. Herzberg^49^ calculated the compositions of melts produced by fertile peridotite, and the composition of olivine fractionated from those melts (Supplementary Fig. S5a). The olivine Ni contents (Supplementary Fig. S5a) of MORB overlap with the calculated composition of olivine fractionated from melts of fertile peridotite, consistent with a peridotite-dominated mantle source for MORB. Olivine within Site U1431 basalts has higher Ni compared with average MORB and deviates significantly from the calculated composition of olivine fractionated from fertile peridotite melts (Supplementary Fig. S5a). These olivines are similar to those of Hawaiian and Hainan OIB, indicating that Site U1431 basalts were produced by melting of a mantle source that contained significant amounts of recycled oceanic crust (e.g., garnet pyroxenite)^10,48^. In contrast, olivine within the basalts of Site U1500 has low Ni contents, similar to average MORB and the calculated composition of olivine fractionated from melts of fertile peridotite (Supplementary Fig. S5a).

The flat N-MORB-like primitive-mantle-normalized heavy REE pattern (Supplementary Fig. S3) further confirms a spinel peridotite mantle source. Then we can model the batch melting of a hypothetical mantle source in the spinel or garnet stability field by La/Yb-Sm/Yb compositions (Supplementary Fig. S7). Notably, for a given La/Yb, melting of garnet peridotite generates higher Sm/Yb ratios (Supplementary Fig. S7). In comparison, all of Site U1500 basalts and most of Pacific and Indian Ocean MORB are located into the melting trend of spinel peridotite (Supplementary Fig. S7). Thus, the observed variations in the strongly incompatible element contents (Supplementary Fig. S3), as well as the good correlations between Nb and a range of incompatible elements (Supplementary Fig. S4), record the effect of olivine fractionation or variable degrees of partial melting of the spinel peridotite source.

### Mantle potential temperature during initial spreading of the SCS

Models of mantle plumes based on the petrology and geochemistry of OIB require a heterogeneous mantle and a thermal anomaly^49–51^. Mantle potential temperatures (*T*p) are used to constrain the magnitude of the thermal anomaly through comparisons between the *T*p of OIB and MORB. The *T*p is the temperature that the mantle would have if it were raised adiabatically to Earth’s surface without melting^52^. In the thermal plume model, active thermal upwelling produces anomalously hot mantle, characterized by a non-zero excess temperature (*T*_ex_ = *T*_p_^h^°^tsp^°^t^ − *T*_p_^MOR^). Most OIB have *T*_ex_ values higher than those of MORB, with mean *T*_ex_ values of 175–195 °C^51^. Therefore, thermally driven mantle plumes are common^51^, and *T*p can be used to determine the thermal state of the mantle and recognize the presence of mantle plumes.

**Figure 5:**
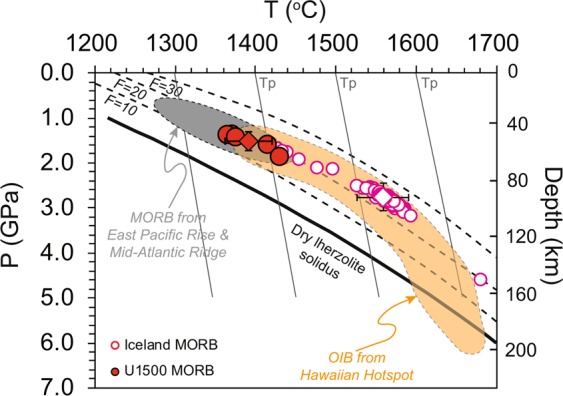
Melting temperatures and pressures calculated for Site U1500 basalt samples. Lherzolite solidus and melt fraction isopleths are from Katz *et al*.^63^. Curved lines represent melting adiabats, near-vertical lines (grey) represent solid mantle adiabats, which are modified from Lee *et al*.^50^. Hawaiian hotspot source regions for shield and post-shield basalts are modified from Lee *et al*.^50^. Temperatures and pressures calculated for MORB from the East Pacific Rise and Mid-Atlantic Ridge are modified from Lee *et al*.^50^, either. MORB from Iceland are derived from Petrological Database (http://www.earthchem.org/petdb) and calculated results are shown in Supplementary Table S3. During our calculations and for our samples, only fresh basalts with MgO > 6.75 wt.% were used. All magma compositions were corrected for olivine-fractionation up to olivine Mg# of 0.9 (molar Mg^2+^/(Mg^2+^ + Fe^2+^)).

The *T*p can be calculated from the temperature of partial melting of the mantle which are derived from the calculated temperature of a primitive melt^50,51,53^. Most basalt thermometers are based on the assumption of a peridotite mantle source and require that melt compositions are close to those of primitive melts or underwent fractionation of olivine only. The olivine–liquid thermometer (*T*°^l-liquid^) does not require a specific lithology in the mantle source. In this study, we used the SiO_2_-based thermobarometer^50^, olivine-liquid equilibria based thermometer^51^, and the mass balance based inverse model of PRIMELT3 MEGA software^53^ to estimate temperature of partial melting of mantle and then *T*p. Samples affected by seawater alteration and those that recorded extensive fractional crystallization (MgO <6.75 wt.%) were excluded. Details of how the temperatures were calculated can be referred in Supplementary Materials. Melting temperature as calculated from SiO_2_-based thermobarometer is 1390 ± 29 ^o^C (1 standard deviation (SD)), melting temperature as calculated from olivine-liquid equilibria ranges from 1390 ^o^C to 1450 ^o^C (Supplementary Fig. S8), and melting temperature as calculated from PRIMELT3 MEGA software is 1367 ± 38 ^o^C. We further estimated *T*p for Site U1500 basalts by SiO_2_-based thermobarometer and PRIMELT3 MEGA software, the average results of which vary from ~1380 °C to ~1450 °C (Fig. 5 and Supplementary Table S1). The consistency of the partial melting temperature and *T*p values as calculated by different methods increases confidence in the validity of the calculations (Supplementary Table S1).

We compared the results for the basalts of Site U1500 with the calculated *T*p of Iceland MORB, the global MORB array, and Hawaiian OIB (Fig. 5) by using SiO_2_-based thermobarometer. The advantage of using this method is that it can provide the calculations of temperature and pressure at the same time. Compared with PRIMELT3 MEGA software, SiO_2_-based thermobarometer considers the influence of physical factors during *T*p estimation. In Fig. 5, the MORB *T*p array represents the background upper-mantle temperature, whereas the *T*ps of the Iceland and Hawaii samples represent plume-affected mantle. The estimated *T*p value of the basalts from Site U1500 is around 1380 °C, falling into zone of global MORB (Fig. 5). The melting temperature of basalts from Iceland is 1417–1593 °C (Supplementary Table S3), with a mean of 1558 ± 32 °C (1 SD), the estimated *T*p of which is close to the value of ~1530 °C calculated for Hawaiian OIB (Fig. 5). Thus, in summary, the *T*p calculated from Site U1500 basalts is lower than the *T*ps of basalts from Iceland and Hawaii and close to normal MORB (Fig. 5). There was no thermal anomaly in the upper mantle when spreading of the SCS began.

## Implications

The geochemical data indicate that spinel peridotite melting produced Site U1500 basalts. The *T*p values show that the upper mantle that produced Site U1500 basalts had a similar thermal state to that of MORB-producing upper mantle. These conditions are significantly different from those inferred for basalts from Site U1431, which record a pyroxenite source and the presence of a thermal anomaly at the end of spreading^10^. Therefore, geochemical features attributable to plumes were absent during the initial stages of SCS spreading and increased towards the end of spreading^10,13,14^, meaning that the effects of mantle plumes can be neglected during initial spreading.

The geochemistry and *T*p of MORB and OIB from the SCS can be used to constrain the evolution of the Hainan plume. The ~25 Ma MORB of the Kenting Mélange, southern Taiwan, have a depleted mantle-like isotopic composition, indicating that the Hainan plume did not contribute to the formation of oceanic crust at this time^13^. In contrast, the crystallization temperatures of 16 Ma basalts from Site U1431 are higher than those of N-MORB^12^. The geochemical and isotopic characteristics of Site U1431 basalts indicate that their mantle source was pyroxenite that included an enriched EM2-like component^14^. Therefore, plume activity was exerting a strong effect on the SCS by 16 Ma. Ocean island basalts continued to form within the SCS basin and Hainan area after the cessation of SCS spreading (~15 Ma to present)^17,19,27,54,55^. The mantle source of these OIB is characterized by a thermal anomaly; the *T*p of SCS OIB is 1647–1688 °C and the *T*p of Hainan OIB is 1468–1582 °C^9,17^, and their Sr, Nd, and Pb isotope ratios record mixing between two end-members, one of which is an EM2-like component^18^. These data indicate that the Hainan plume affected the upper mantle after ~25 Ma and that plume–ridge interaction did not begin until >8 Myr after the initial spreading.

**Figure 6:**
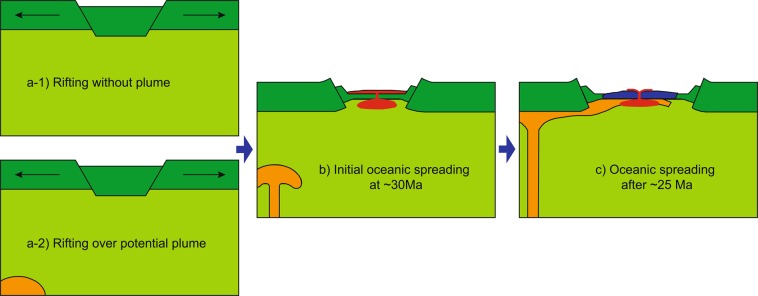
Cartoon showing the mantle dynamics of the SCS basin from continental rifting to oceanic crustal spreading. (a-1) Continental rifting without mantle plume activities in the mantle. (a-2) Continental rifting with potential mantle plume nearby but no influence. (**b**) In a scenario of rifting induced by tectonic forces, volcanism is the result of decompressing melting of the upwelling asthenosphere and postdates the main onset of rifting. Partial melting of the upper mantle might cause the suction of upper mantle and finally triggered the upwelling of deep hot materials or accelerated the upwelling of existing mantle plume nearby. (**c**) After transform from rifting to spreading, plume materials could be guided towards the thinning lithosphere (upside-down drainage of Sleep^57^) causing plume-ridge interaction model after ~25 Ma. The cartoon is modified from Buiter and Torsvik^64^.

We propose that the mantle beneath the SCS basin evolved as follows: (1) Prior to ~33 Ma, tectonic forces caused rifting and continental breakup without a mantle plume (Fig. 6a-1) or in the vicinity of an upwelling plume from the deep mantle (Fig. 6a-2). (2) At ~33 Ma, rifting evolved into initial spreading. Partial melting of the upper mantle might have caused upwelling of deep hot material or accelerated upwelling of an existing mantle plume (Fig. 6b). (3) At ~25 Ma, plume material began to interact with the thinning lithosphere in response to a reversal of flow patterns caused by spreading of the basin^56,57^ (Fig. 6c). Consequently, the rocks record plume–ridge interaction after ~25 Ma, indicating that plate tectonics, rather than a mantle plume, caused the continental rifting and initial spreading that formed the SCS. The primary cause of continental breakup and rapid transition to subsequent initial spreading was probably westward subduction and retreat of the Pacific Plate^58^. However, the mantle plume might have been a deep expression of continental breakup. If so, then the SCS could provide an excellent opportunity for research into rifting-induced mantle plumes.

## Methods

Samples were first crushed into gravel-size chips. Clean chips were then pulverized in a corundum mill. Major element compositions of whole rocks were determined using a Thermo Scientific ARL 9900 X-ray fluorescence spectrometer (XRF) at the State Key Laboratory for Mineral Deposits Research, Nanjing University, China. Measurements of bulk rock trace element concentrations were completed at the Department of Geology, Northwest University, China. Trace elements were determined using an ELANG100DRC inductively coupled plasma mass spectrometer (ICP-MS) after the acid digestion (HF + HNO_3_) conducted in Teflon bombs. Trace elemental analyses of the USGS rock standards (BHVO-2, BCR-2, and AGV-2) are reported for comparison. Major element analysis and BSE imaging of minerals were carried out by EPMA (JEOL JXA-8230) equipped with four wavelength-dispersive spectrometers at the State Key Laboratory of Marine Geology, Tongji University. Details of methods are provided in the Supplementary Materials.

## Supplementary information

Supplementary Information.
