# Supplementary material for: Non-mantle-plume process caused the initial spreading of the South China Sea
Source: Sci Rep. 2020 May 22;10:8500. doi: 10.1038/s41598-020-65174-y (PMC7244718; doi:10.1038/s41598-020-65174-y)
Supplement: Supplementary file 1 — Supplementary Information. [file 41598_2020_65174_MOESM1_ESM.docx]

Supplementary Materials for

**Non-mantle-plume process caused the initial spreading of the South China Sea**

Xun Yu*, Zhifei Liu*

State Key Laboratory of Marine Geology, Tongji University, Shanghai 200092, China

Corresponding author: yuxun@tongji.edu.cn

**Contents of this file**

**Methods**

**Temperature calculation**

**Figures S1 to S8**

**Tables S1 to S3**

**References**

Methods

New whole rock major and trace elemental data for basalts from Site U1500 are presented in this study. Samples were first crushed into gravel-size chips. Clean chips were then pulverized in a corundum mill. Major elemental compositions of whole rocks were determined using a Thermo Scientific ARL 9900 X-ray fluorescence spectrometer (XRF) at the State Key Laboratory for Mineral Deposits Research, Nanjing University, China. According to the measured values of standards (GBW-07103 and GBW-07105), the analytical accuracy is about ± 1% for elements with concentrations >1.0 wt.% and about ± 10 wt.% for elements with concentrations <1.0 wt.%. The results of diverse standards (BHVO-2 and BCR-2) suggest the uncertainties are less than ± 1% for elements Si, Ti, Al, Fe, Mn, Mg, Ca, K, and P, and about ± 5% for Na (Supplementary Table S1). Measurements of bulk rock trace elemental concentrations were completed at the Department of Geology, Northwest University, China. Trace elements were determined using an ELANG100DRC inductively coupled plasma mass spectrometer (ICP-MS) after the acid digestion (HF+HNO_3_) conducted in Teflon bombs. Analyses of the USGS rock standards (BHVO-2, BCR-2, and AGV-2; reference data are from Jochum *et al*.^1^) indicate the precision and accuracy are better than 5% for Sc, V, Co, Ni, Rb, Sr, Y, Zr, Nb, Ba, Ta, Th, U and REEs, and 10% for Cr, Cs, Hf, and Pb (Supplementary Table S1). In order to understand the genesis of Site U1500 basalts, data of samples from Site U1431 were collected to make the comparison. Basalts from Site U1500 stand for the oceanic crust formed in the early stage of seafloor spreading while basalts from Site U1431 stand for the oceanic crust formed in the end of seafloor spreading. The comparison of them can help us understand the formation of the SCS.

Major element analysis and BSE imaging of minerals were carried out by EPMA (JEOL JXA-8230) equipped with four wavelength-dispersive spectrometers at the State Key Laboratory of Marine Geology, Tongji University, China. The EPMA was operated at an accelerating voltage of 15 kV and with a beam current of 10 nA, a 5 μm beam size and a 30 s counting time^2^. Natural mineral standard (SPI) was used to calibrate all quantitative analyses and a ZAF correction was used for data reduction. In the measurement, the average NiO result of SPI is 0.36±0.05 (1standard error; reference data is 0.37) while the average FeO result of SPI is 7.22±0.08 (1standard error; reference data is 7.25). The average MgO result of SPI is 50.4±0.4 (1standard error; reference data is 50.97). The results show that uncertainties are less than ± 5% for NiO, less than ± 2% for MgO, and less than ± 1% for FeO. All data including standard are shown in Supplementary Table S2.

Temperature calculation

SiO_2_-based thermobarometer is less sensitive to variations in mantle composition because silica, being a major element, is buffered at a given temperature and pressure by the mineralogy of the system^3^. The main advantages of SiO_2_-based thermobarometer are relative low uncertainty and much simpler to be implemented. The uncertainty of barometer is of ±0.20 GPa and the uncertainty in temperature is ±3% of a temperature result. Successful application of SiO_2_-based thermobarometer in the mantle requires knowledge of the primary magma composition, that is, the composition of the magma when it was last in equilibrium with the mantle. The other requisite is both olivine and orthopyroxene be present in the mantle source. Therefore, during the calculation, only samples with MgO > 6.75 wt.% were used. Olivine increments in equilibrium with the instantaneous melt composition, assuming compositionally dependent olivine/melt K_D_(Fe/Mg), are back-added to the magma until the magma composition reached equilibrium with a mantle composition. A VisualBasic Excel Macro for fractionation correction and P-T calculation was used during our calculation. In calculation, we assumed a residual mantle composition equivalent to an olivine having an Mg#=Mg/(Mg+Fe^2+^)=0.9. The melting temperature derived from our samples is 1390±29 ^o^C and the pressure is 1.5±0.2 GPa. Finally, primary magma T and P we calculated were used to infer mantle potential temperatures in Figure 5 in the main text. Results for our samples are shown in Supplementary Table S1.

PRIMELT software was first developed by Herzberg and O’Hara^4^ and was updated several times until to latest PRIMELT3 MEGA.XLSM version, which is a mass balance solution to the primary magma problem for an assumed peridotite composition^5^. PRIMELT is calibrated from experiments in peridotite KR4004^6^ and parameterizations of these and other experiments^4^. PRIMELT reconstructs the primary magma composition by addition or subtraction of olivine to the lava composition. In our study, we used the PRIMELT3 MEGA.XLSM version to calculate the melting temperatures. PRIMELT3 MEGA.XLSM computes the composition of olivine in equilibrium with liquid in the inverse model. This requires knowledge of the Fe-Mg partitioning between these phases, also called K_D_(Fe-Mg), and the method of Toplis^7^ was adopted. During the calculation, only samples with MgO > 6.75 wt.% were used. The olivine liquidus temperature we got is 1367±38 ^o^C. PRIMELT3 MEGA.XLSM can provide the estimation of mantle potential temperature directly by the equation of Tp=1025+28.6MgO-0.084MgO^2^ without concerning the influence of physical parameters like melt fraction, heat of fusion, and heat capacity. The accurate of the calculated mantle potential temperature has been reported within ±42 ^o^C^5^. The mantle potential temperature of our samples is 1450±52 ^o^C. Results for our samples are shown in Supplementary Table S1.

Olivine-liquid equilibria^8^ is also useful for T estimation because the ratio (X_Fe_/X_Mg_)^ol^/(X_Fe_/X_Mg_)^liq^ (or K_D_(Fe-Mg)^ol-liq^) is nearly constant over a wide range of temperatures, bulk compositions and fO_2_ conditions^9^, and the ratio X^ol^_Mg_/X^liq^_Mg_ (K_d_(Mg)) is highly sensitive to temperature (X_Fe_ and X_Mg_ are cation fractions of Fe and Mg, respectively). Because the K_d_(Fe)^ol-liq^ is close to 1, X^liq^_Fe_ of primitive liquids can be estimated more precisely than X^liq^_Mg_, provided that whole rock or glass compositions can be traced to an olivine control line. Thus, if we know the Mg and Fe compositions of olivine, and Fe composition of bulk rock, then we can estimate the composition of Mg. As a result, the melting temperature can be discovered. In this study, the FeO^total^ of basalt samples with MgO higher than 6.75 wt.% ranges from 8 wt.% to 9 wt.%. Then, we selected FeO^total^ is equal to 8 wt.% and 9 wt.% as lower and upper limits in the calculation. The Fo value we selected here is 83 according to the measurement of olivine (Supplementary Table S2). The K_D_(Fe-Mg) we set in calculation is 0.32. With the help of elemental data of olivines, we can estimate the values of X^liq^_Mg_ and X^liq^_Fe_ of more primitive basalt melts. After we got above values, we can plot them on the figure of olivine saturation surface after Roeder and Emslie^9^ and Langmuir and Hanson^10^. Figure of olivine saturation surface can be referred to Putirka^8^. Melting temperature of mantle source then can be estimated. The temperature calculated from lower limit is about 1390 ^o^C while the temperature calculated from upper limit is about 1450 ^o^C. Thus the temperature of our samples ranges from 1390 ^o^C to 1450 ^o^C.


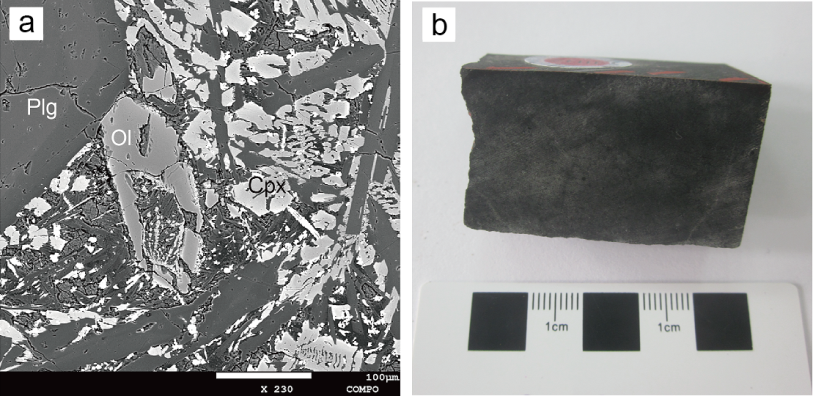


Figure S1. Primary rock-forming minerals and textures of basalt (a) and representative photograph of fresh basalt sample in this study (b) from Site U1500. (a) Backscattered electron image of basalt sample with olivine phenocryst. At the microscopic scale, basalt sample shows a well-preserved primary igneous mineral assemblage (olivine, plagioclase, clinopyroxene, and Fe-Ti oxides) and microtextures (e.g., aphyric). Elongated prismatic subhedral plagioclase is the dominant phenocryst mineral (1%-35%; 1-20 mm in size), and olivine remains as an occasional phenocryst mineral (≤ 10%; 0.5-2.5 mm in size). Rare clinopyroxene phenocrysts are identified.


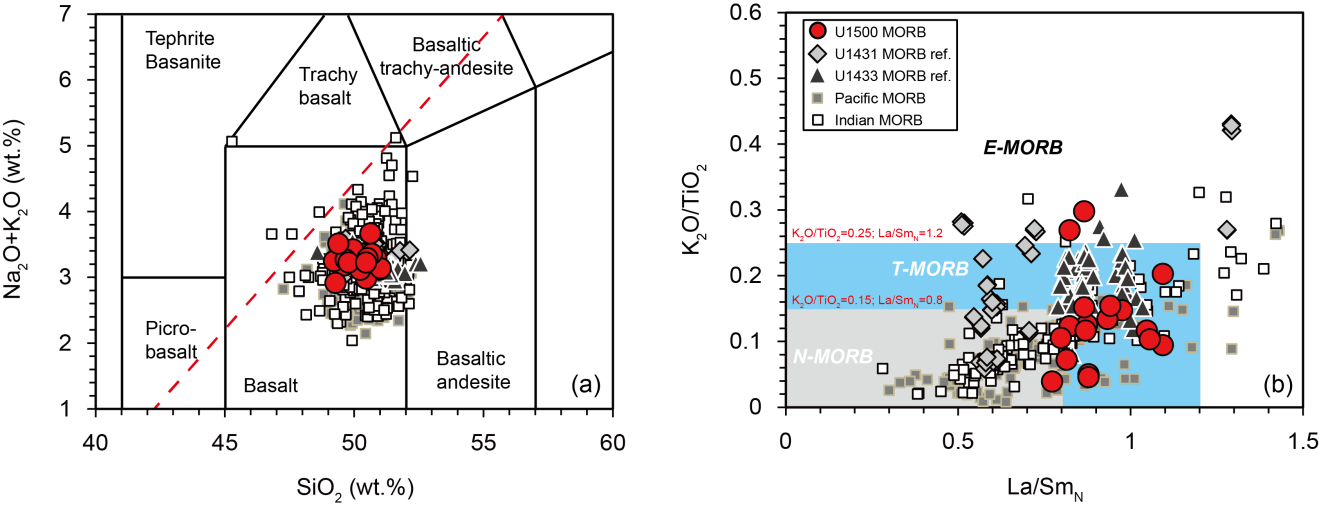


Figure S2. Plots of SiO_2_ versus Na_2_O+K_2_O (a) and La/Sm_N_ versus K_2_O/TiO_2_ (b) for Site U1500 basalt samples. Reference data for basalt samples from Sites U1431 and U1433 are shown for comparison^11,12^. Data for global MORB (Pacific Ocean MORB, and Indian Ocean MORB) are derived from Petrological Database (http://www.earthchem.org/petdb). N here means primitive mantle normalized. The primitive mantle values are from McDonough and Sun^13^.


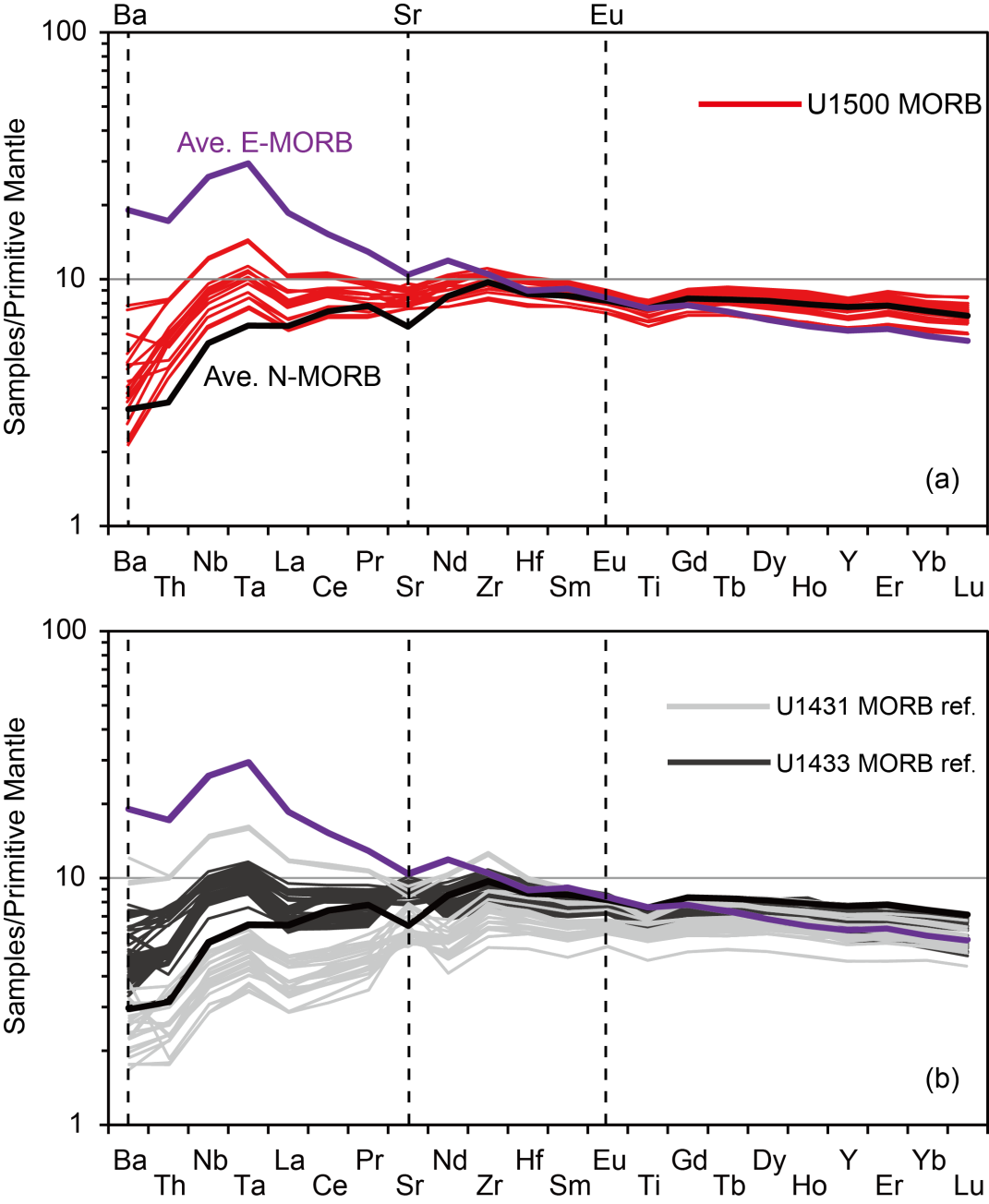


Figure S3. Plots of primitive mantle normalized whole rock trace element abundances for (a) Site U1500 and (b) Sites U1431 and U1433 basalt samples. Average trace elemental data for N-MORB and E-MORB are from Gale *et al*.^14^. Reference data for basalt samples from Sites U1431 and U1433 are referred to Zhang *et al*.^11,12^. The primitive mantle values are from McDonough and Sun^13^.


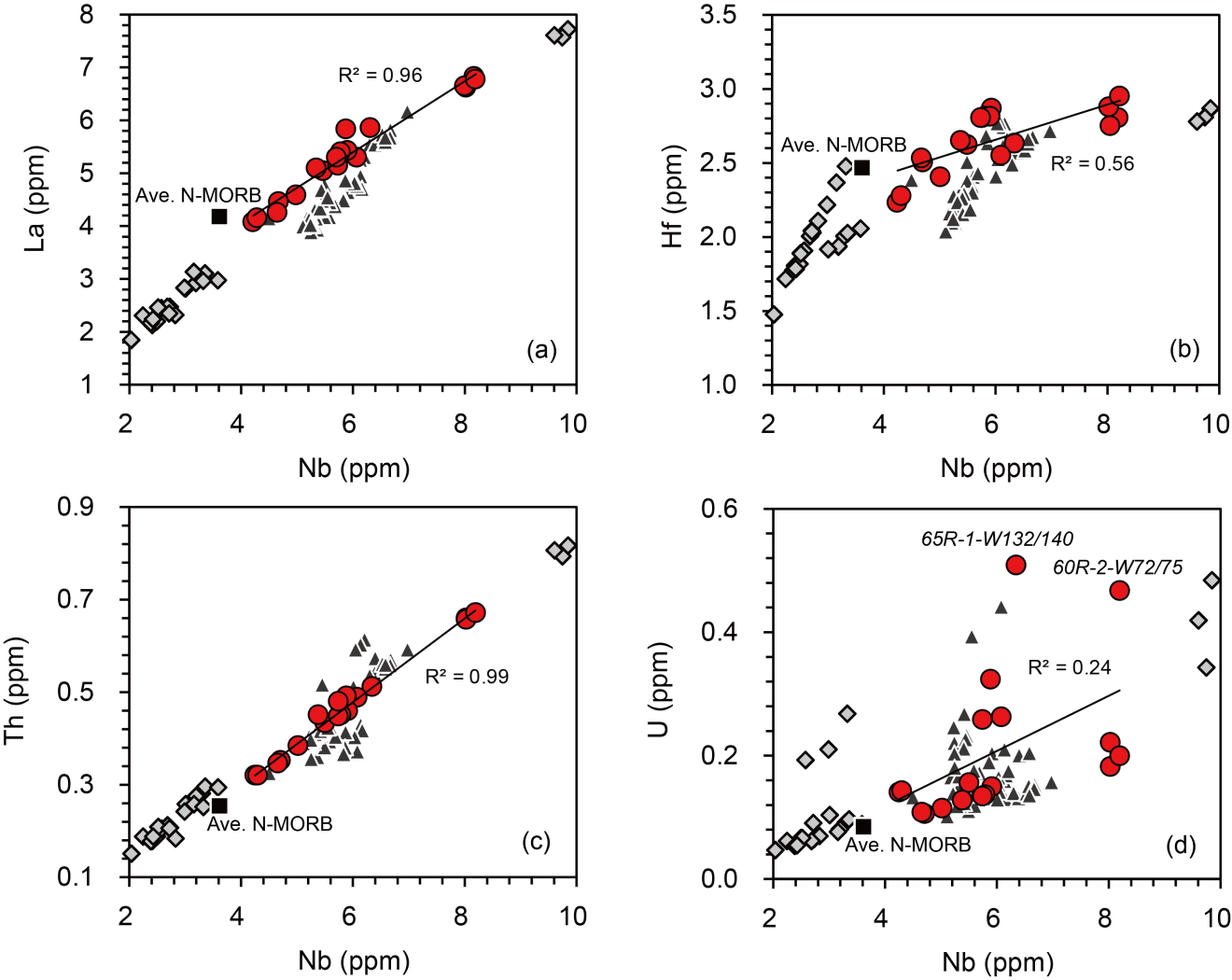


Figure S4. Plots of La (a), Hf (b), Th (c), and U (d) versus Nb for Site U1500 basalt samples. Reference data for MORB samples from Sites U1431 and U1433 are referred to Zhang *et al*.^11,12^. Average trace elemental data for N-MORB is from Gale *et al*.^14^. Symbols are the same as in Supplementary Figure S2.


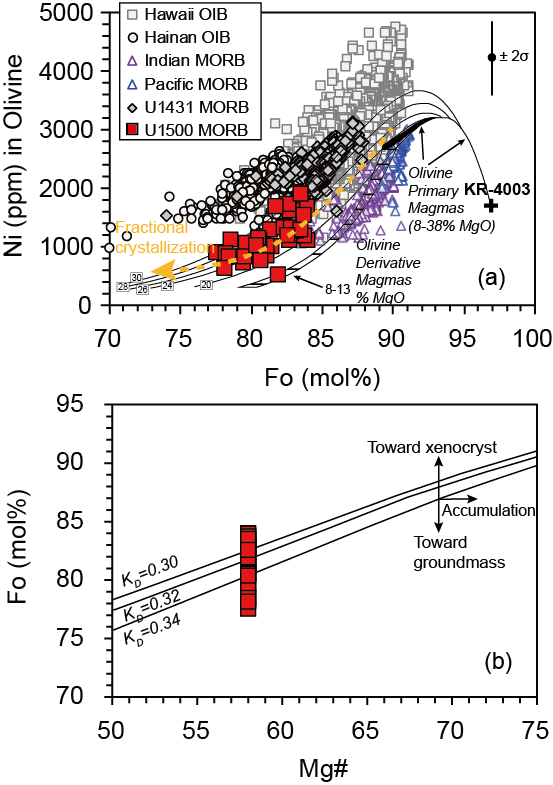


Figure S5. Variations in olivine Mg number (Fo) versus Ni (a) and whole-rock Mg# versus Fo content of olivine (b). In plot (a), the calculated olivine compositions are based on Herzberg^15^. Black area shows the olivines of primary magmas (MgO of 8-38 wt.%) from fertile peridotite KR-4003 with 1964 ppm Ni, 1007 ppm Mn, and 8.02 wt.% FeO. Numbered lines are calculated olivines of olivine-fractionated derivative magmas, and the numbers indicate the MgO contents of olivine derivative magmas. Short line with end-bars in (a) is ±2σ Ni variation of the primary magma from which olivines crystallize. Data of olivines for global MORB (Indian ridge and East Pacific Rise) and Hawaiian OIB are from Sobolev *et al*.^16^. Data of olivines for Hainan OIB are from Liu *et al*.^17^. Data of olivines for U1431 MORB are from Zhang *et al*.^11^. The fractional crystallization trend is from Sato^18^. In plot (b), Mg# = 100Mg/(Mg+Fe^2+^) calculated assuming Fe^2+^/Fe_total_ = 0.9 for whole-rock and total Fe as Fe^2+^ in olivine.


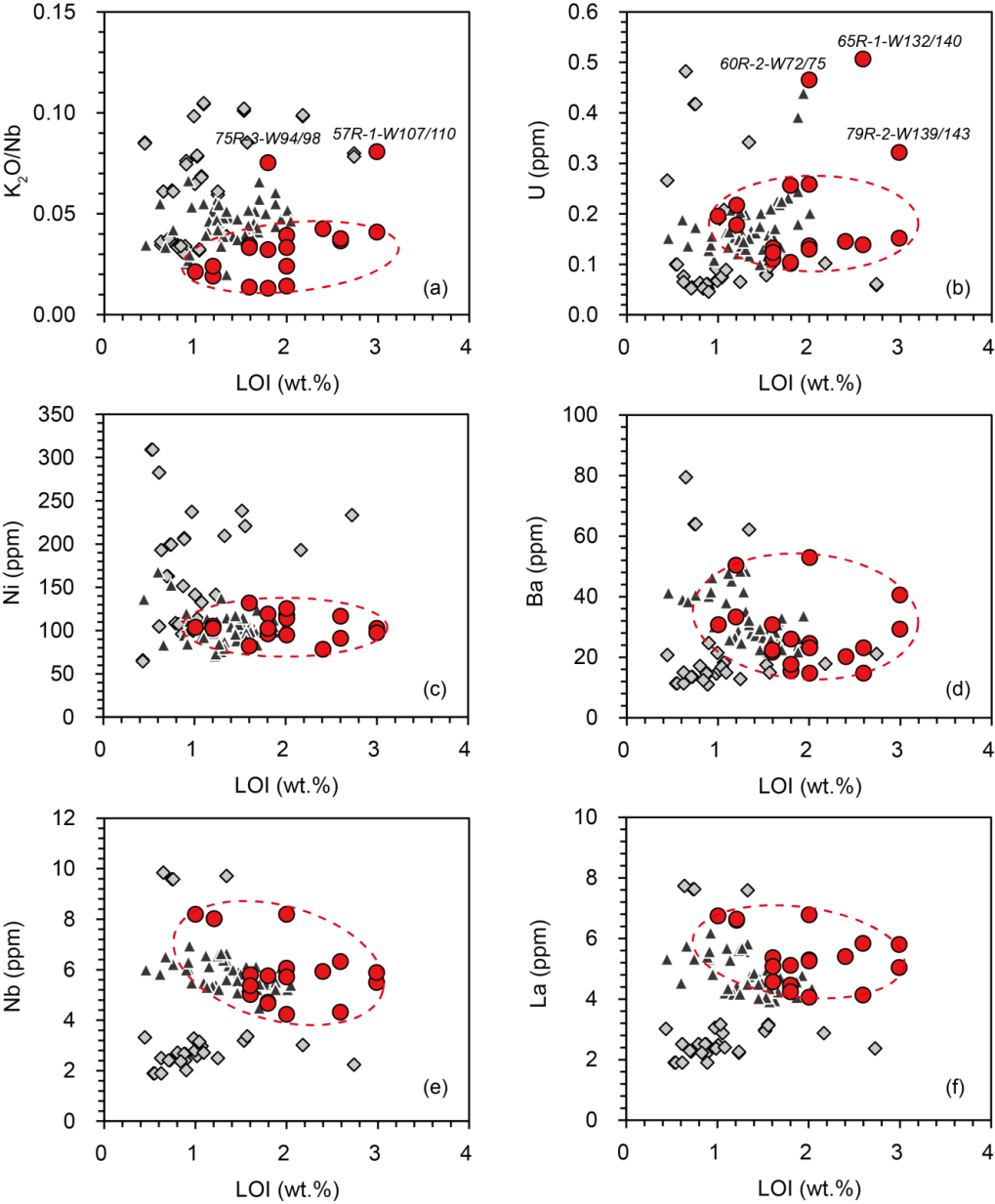


Figure S6. Plots of K_2_O/Nb (a), U (b), Ni (c), Ba (d), Nb (e), and La (f) versus LOI (wt.%) for Site U1500 basalt samples. Reference data for basalt samples from Sites U1431 and U1433 are shown for comparison^11,12^. Symbols are the same as in Supplementary Figure S2.


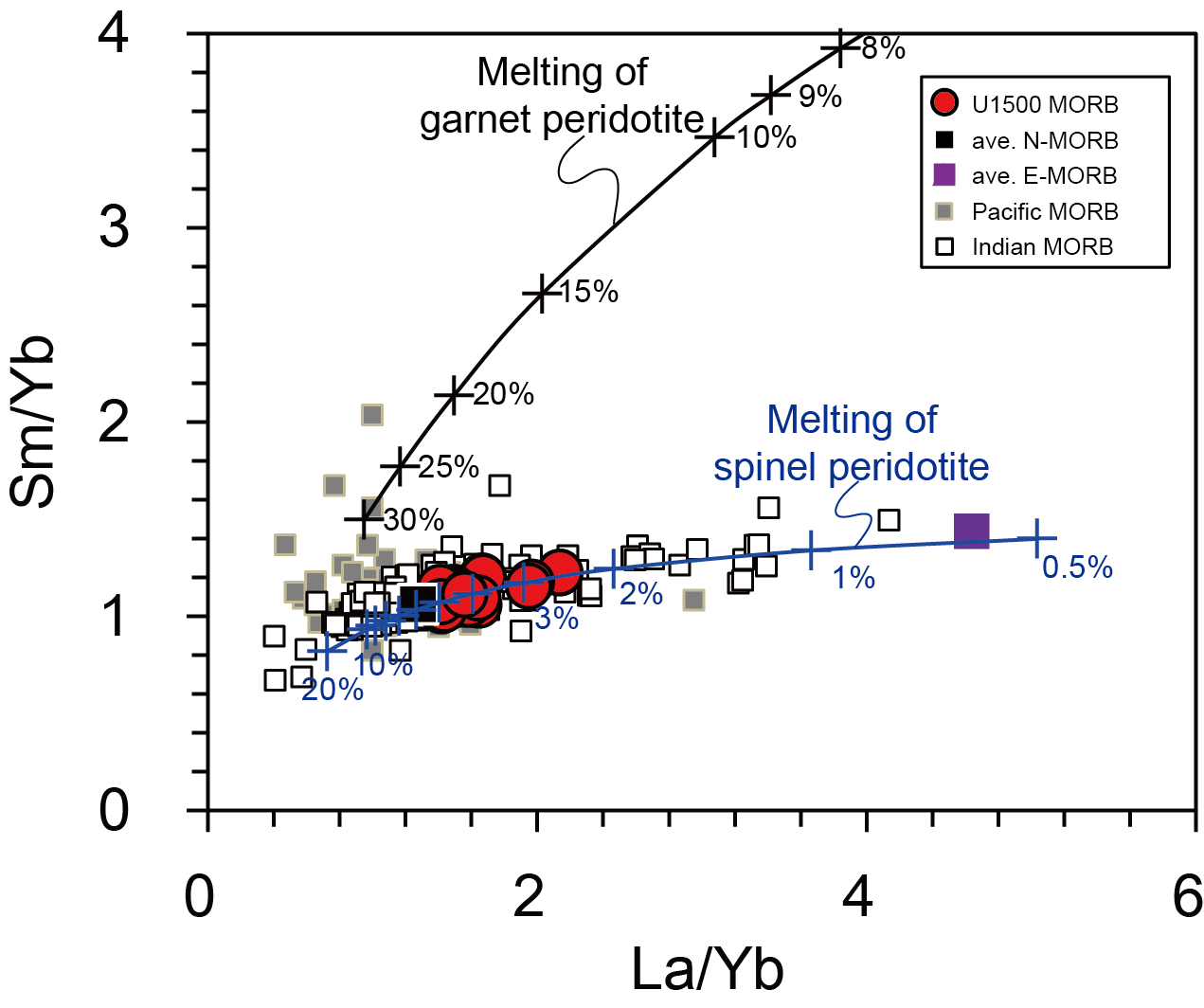


Figure S7. Plot of Sm/Yb vs. La/Yb. Also shown are batch melting curves calculated for spinel peridotite and garnet peridotite. The numbers in the plot represent the degrees of partial melting. Partition coefficients can be referred to Johnson *et al*.^19^. The starting material for garnet peridotite is ol, 62%; opx, 15%; cpx, 15%; gt, 8%; starting material for spinel peridotite is ol, 50%; opx, 15%; cpx, 15%; sp, 20%; melting reaction in garnet field: ol, 4%; opx, 4%; cpx, 70%; gt, 22%; melting reaction in spinel field: ol, 10%; opx, 0%; cpx, 60%; sp, 30%. The starting elemental abundance can be referred to Workman *et al*.^20^. Average elemental data for N-MORB and E-MORB are from Gale *et al*.^14^. Data for global MORB (Pacific Ocean MORB, and Indian Ocean MORB) are derived from Petrological Database (http://www.earthchem.org/petdb).


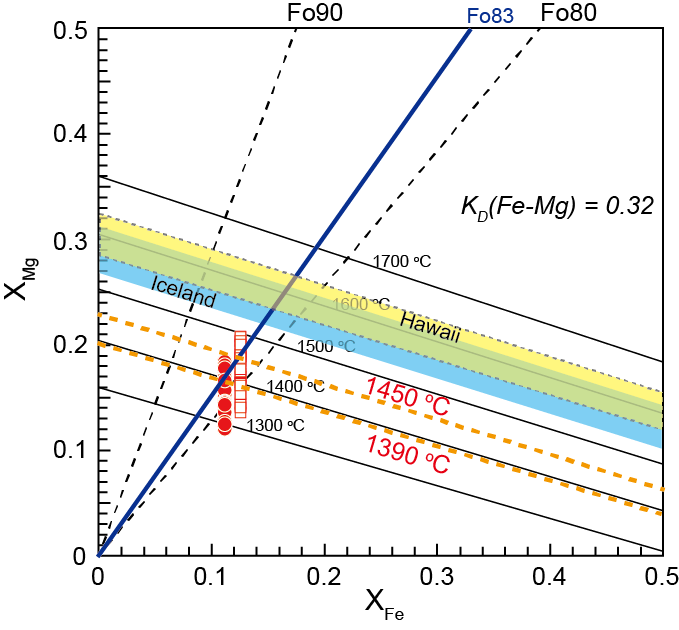


Figure S8. Plot of olivine saturation temperature for sample 67R-2-W68/71. Olivine saturation surface is after Roeder and Emslie^9^ and Langmuir and Hanson^10^. X_Mg_ and X_Fe_ are cation fractions of Mg and Fe, respectively. Thermometer of olivine-liquid equilibria can be referred to Putirka^8^. During calculation, we set FeO^total^ of basalt sample is equal to 8 wt.% and 9 wt.% as lower and upper limits. Red circles stand for results derived from lower limit (FeO^total^ = 8 wt.%), while white squares stand for results derived from upper limit (FeO^total^ = 9 wt.%). Temperature results of Hawaii OIB and Iceland MORB can be referred to Putirka^8^. Details of calculation can be found in Supplementary Temperature calculation.

Table S1. Major and trace elemental compositions of IODP Expedition Site U1500 basalts and international standards.

Table S2. Major elemental compositions of olivines from sample 67R-2-W68/71 and standard.

Table S3. Major elemental compositions and calculated melting temperature and pressure for basalts with MgO > 6.5 wt.% from Iceland.

| **Table S1. Major and trace elemental compositions of IODP Expedition Site U1500 basalts and international standards** | | | | | | | |
| --- | --- | --- | --- | --- | --- | --- | --- |
|  | 17SCS00-02 | 17SCS00-05 | 17SCS00-10 | 17SCS00-11 | 17SCS00-14 | 17SCS00-15 | 17SCS00-18 |
| Sample Name | 57R-1-W107/110 | 58R-2-W64/66 | 60R-2-W72/75 | 60R-3-W89/93 | 63R-2-W27/30 | 63R-2-W67/70 | 65R-1-W132/140 |
| Top depth | 1379.87 | 1390.11 | 1409.96 | 1411.63 | 1419.9 | 1420.3 | 1434.14 |
| Bottom depth | 1379.9 | 1390.13 | 1409.99 | 1411.66 | 1419.93 | 1420.33 | 1434.17 |
| *Major element (wt.%)* | |  |  |  |  |  |  |
| SiO2 | 49.51 | 50.25 | 49.53 | 50.45 | 49.16 | 49.51 | 48.23 |
| TiO2 | 1.479 | 1.355 | 1.568 | 1.555 | 1.623 | 1.642 | 1.541 |
| Al2O3 | 16.69 | 16.41 | 16 | 15.46 | 16.55 | 16.46 | 15.22 |
| Fe2O3 total | 8.55 | 9.08 | 10.01 | 10.14 | 9.82 | 9.58 | 11.7 |
| FeO total | 7.695 | 8.172 | 9.009 | 9.126 | 8.838 | 8.622 | 10.53 |
| MnO | 0.232 | 0.125 | 0.144 | 0.151 | 0.193 | 0.167 | 0.185 |
| **MgO** | **5.64** | **7.3** | **7.01** | **7.04** | **6.65** | **6.51** | **6.87** |
| CaO | 11.9 | 10.83 | 10.63 | 10.87 | 11.66 | 11.4 | 10.82 |
| Na2O | 3.15 | 3.08 | 2.99 | 2.93 | 2.96 | 3.08 | 2.95 |
| K2O | 0.44 | 0.17 | 0.32 | 0.15 | 0.19 | 0.17 | 0.23 |
| P2O5 | 0.171 | 0.161 | 0.216 | 0.2 | 0.205 | 0.209 | 0.203 |
| LOI | 2.99 | 1.6 | 2 | 1.2 | 1.2 | 1 | 2.59 |
| TOTAL | 100.76 | 100.35 | 100.43 | 100.15 | 100.21 | 99.72 | 100.54 |
| *Trace element (ppm)* | |  |  |  |  |  |  |
| Li | 15.7 | 15.3 | 20.1 | 8.60 | 15.6 | 11.2 | 21.3 |
| Be | 0.54 | 0.53 | 0.64 | 0.61 | 0.59 | 0.60 | 0.67 |
| Sc | 35.4 | 32.2 | 34.9 | 34.8 | 35.6 | 36.4 | 38.7 |
| V | 249 | 231 | 271 | 260 | 269 | 275 | 279 |
| Cr | 292 | 286 | 263 | 266 | 258 | 264 | 253 |
| Co | 44.4 | 38.5 | 41.1 | 38.8 | 40.8 | 40.4 | 34.5 |
| Ni | 101 | 132 | 94.0 | 104 | 101 | 104 | 89.7 |
| Cu | 33.2 | 71.0 | 71.3 | 73.6 | 70.2 | 75.6 | 60.1 |
| Zn | 81.3 | 69.1 | 77.8 | 78.7 | 80.3 | 81.3 | 82.0 |
| Ga | 16.6 | 15.4 | 16.4 | 16.1 | 16.8 | 17.2 | 16.6 |
| Ge | 1.42 | 1.56 | 1.54 | 1.63 | 1.52 | 1.30 | 1.71 |
| Rb | 4.47 | 2.34 | 5.06 | 2.12 | 3.50 | 3.12 | 3.98 |
| Sr | 193 | 166 | 187 | 178 | 180 | 187 | 160 |
| Y | 30.2 | 27.8 | 32.0 | 31.0 | 33.4 | 33.9 | 35.0 |
| Zr | 104 | 94.2 | 111 | 109 | 113 | 116 | 101 |
| Nb | 5.48 | 5.00 | 8.18 | 8.03 | 8.02 | 8.20 | 6.33 |
| Cs | 0.077 | 0.11 | 0.35 | 0.12 | 0.15 | 0.17 | 0.10 |
| Ba | 40.2 | 30.4 | 52.6 | 50.1 | 33.1 | 30.6 | 23.0 |
| La | 5.05 | 4.58 | 6.81 | 6.62 | 6.65 | 6.77 | 5.86 |
| Ce | 14.5 | 13.0 | 17.6 | 17.3 | 17.5 | 17.9 | 15.1 |
| Pr | 2.12 | 1.91 | 2.48 | 2.41 | 2.47 | 2.52 | 2.24 |
| Nd | 11.4 | 10.3 | 12.9 | 12.4 | 12.9 | 13.1 | 11.9 |
| Sm | 3.66 | 3.29 | 3.90 | 3.79 | 3.97 | 4.01 | 3.77 |
| Eu | 1.31 | 1.18 | 1.35 | 1.31 | 1.36 | 1.40 | 1.33 |
| Gd | 4.46 | 4.04 | 4.68 | 4.59 | 4.82 | 4.93 | 4.76 |
| Tb | 0.81 | 0.73 | 0.85 | 0.82 | 0.88 | 0.89 | 0.87 |
| Dy | 5.26 | 4.77 | 5.47 | 5.36 | 5.76 | 5.87 | 5.85 |
| Ho | 1.11 | 1.01 | 1.17 | 1.13 | 1.23 | 1.25 | 1.27 |
| Er | 3.21 | 2.91 | 3.37 | 3.28 | 3.57 | 3.64 | 3.73 |
| Tm | 0.48 | 0.43 | 0.50 | 0.48 | 0.53 | 0.54 | 0.56 |
| Yb | 3.03 | 2.74 | 3.19 | 3.10 | 3.38 | 3.47 | 3.56 |
| Lu | 0.45 | 0.41 | 0.47 | 0.46 | 0.51 | 0.52 | 0.54 |
| Hf | 2.62 | 2.41 | 2.81 | 2.75 | 2.88 | 2.95 | 2.63 |
| Ta | 0.37 | 0.33 | 0.54 | 0.53 | 0.53 | 0.54 | 0.42 |
| Pb | 0.72 | 0.42 | 0.68 | 0.70 | 0.73 | 0.70 | 0.60 |
| Th | 0.43 | 0.38 | 0.67 | 0.66 | 0.66 | 0.67 | 0.51 |
| U | 0.15 | 0.11 | 0.47 | 0.18 | 0.22 | 0.20 | 0.51 |
|  |  |  |  |  |  |  |  |
| T-Lee |  | 1370 |  | 1414 |  |  |  |
| P-Lee |  | 1.35 |  | 1.58 |  |  |  |
| T-Herzberg |  | 1349 |  | 1403 |  |  |  |
| Tp-Herzberg |  | 1425 |  | 1502 |  |  |  |
| T-Putirka |  |  |  |  |  |  |  |

|  |  |  |  |  |  |  |  |
| --- | --- | --- | --- | --- | --- | --- | --- |
| 17SCS00-19 | 17SCS00-22 | 17SCS00-23 | 17SCS00-23 re | 17SCS00-26 | 17SCS00-30 | 17SCS00-34 | 17SCS00-35 |
| 65R-2-W9/12 | 67R-2-W68/71 | 69R-1-W14/17 | 69R-1-W14/17 re | 70R-3-W122/126 | 71R-2-W29/32 | 73R-1-W67/70 | 75R-1-W67/71 |
| 1434.41 | 1444.28 | 1452.05 |  | 1460.29 | 1463.29 | 1471.77 | 1481.37 |
| 1434.44 | 1444.31 | 1452.08 |  | 1460.33 | 1463.32 | 1471.8 | 1481.41 |
|  |  |  |  |  |  |  |  |
| 49.02 | 50.01 | 48.43 | 48.42 | 50.51 | 49.77 | 48.69 | 49.22 |
| 1.481 | 1.359 | 1.294 | 1.297 | 1.414 | 1.443 | 1.643 | 1.616 |
| 15.72 | 17.44 | 17.33 | 17.34 | 17.81 | 18.74 | 16.05 | 16.02 |
| 10.2 | 9.11 | 9.36 | 9.38 | 7.47 | 7.1 | 10.49 | 11.39 |
| 9.18 | 8.199 | 8.424 | 8.442 | 6.723 | 6.39 | 9.441 | 10.251 |
| 0.17 | 0.144 | 0.174 | 0.173 | 0.135 | 0.219 | 0.175 | 0.195 |
| **6.85** | **6.25** | **6.48** | **6.43** | **6.66** | **6.7** | **6.22** | **6.09** |
| 11.1 | 11.65 | 12.14 | 12.12 | 11.73 | 11.86 | 11.29 | 10.97 |
| 3.15 | 2.84 | 2.7 | 2.67 | 2.94 | 3.02 | 2.95 | 2.99 |
| 0.2 | 0.1 | 0.16 | 0.16 | 0.15 | 0.06 | 0.25 | 0.19 |
| 0.176 | 0.157 | 0.151 | 0.15 | 0.156 | 0.164 | 0.195 | 0.185 |
| 2 | 2 | 2.59 | 2.59 | 1.8 | 1.8 | 2.4 | 1.6 |
| 100.07 | 101.05 | 100.8 | 100.7 | 100.78 | 100.88 | 100.35 | 100.48 |
|  |  |  |  |  |  |  |  |
| 19.1 | 23.5 | 23.5 | 15.5 | 13.0 | 12.9 | 16.8 | 17.1 |
| 0.54 | 0.47 | 0.49 | 0.48 | 0.48 | 0.33 | 0.60 | 0.62 |
| 37.1 | 30.2 | 30.7 | 31.7 | 34.4 | 34.3 | 39.6 | 39.7 |
| 258 | 222 | 226 | 233 | 248 | 252 | 274 | 268 |
| 236 | 288 | 253 | 266 | 278 | 301 | 218 | 221 |
| 50.1 | 37.7 | 38.0 | 36.8 | 37.6 | 40.7 | 39.7 | 38.2 |
| 116 | 113 | 115 | 102 | 95.9 | 118 | 78.2 | 80.9 |
| 74.7 | 67.7 | 67.3 | 69.2 | 74.0 | 78.8 | 77.8 | 79.6 |
| 79.0 | 70.6 | 71.6 | 70.0 | 76.4 | 75.3 | 83.4 | 82.0 |
| 16.4 | 15.5 | 15.8 | 15.9 | 16.5 | 16.9 | 16.9 | 16.8 |
| 1.35 | 1.32 | 1.58 | 1.31 | 1.52 | 1.33 | 1.67 | 1.70 |
| 3.70 | 3.81 | 3.88 | 2.25 | 2.79 | 0.66 | 5.02 | 3.54 |
| 159 | 153 | 154 | 162 | 155 | 170 | 155 | 154 |
| 31.9 | 27.3 | 27.7 | 28.8 | 30.5 | 29.7 | 36.6 | 36.4 |
| 98.9 | 86.9 | 88.4 | 92.0 | 97.0 | 97.1 | 114 | 112 |
| 6.08 | 4.23 | 4.30 | 4.51 | 4.69 | 4.65 | 5.91 | 5.78 |
| 0.13 | 0.19 | 0.20 | 0.10 | 0.041 | 0.019 | 0.23 | 0.12 |
| 24.4 | 14.4 | 14.5 | 16.4 | 25.6 | 15.3 | 20.2 | 21.5 |
| 5.31 | 4.09 | 4.15 | 4.29 | 4.47 | 4.26 | 5.44 | 5.39 |
| 14.4 | 11.9 | 12.1 | 12.5 | 13.1 | 12.8 | 15.3 | 15.1 |
| 2.06 | 1.79 | 1.82 | 1.88 | 1.98 | 1.96 | 2.26 | 2.23 |
| 11.0 | 9.75 | 9.88 | 10.2 | 10.8 | 10.7 | 12.2 | 12.0 |
| 3.56 | 3.15 | 3.16 | 3.30 | 3.50 | 3.46 | 3.95 | 3.88 |
| 1.27 | 1.13 | 1.14 | 1.17 | 1.25 | 1.24 | 1.37 | 1.35 |
| 4.44 | 3.90 | 3.93 | 4.06 | 4.36 | 4.30 | 4.97 | 4.90 |
| 0.82 | 0.71 | 0.72 | 0.74 | 0.80 | 0.80 | 0.93 | 0.91 |
| 5.49 | 4.67 | 4.70 | 4.91 | 5.23 | 5.17 | 6.16 | 6.10 |
| 1.18 | 1.01 | 1.02 | 1.05 | 1.13 | 1.11 | 1.34 | 1.33 |
| 3.46 | 2.91 | 2.94 | 3.05 | 3.27 | 3.19 | 3.95 | 3.91 |
| 0.51 | 0.43 | 0.43 | 0.46 | 0.49 | 0.47 | 0.59 | 0.58 |
| 3.35 | 2.74 | 2.79 | 2.88 | 3.09 | 3.02 | 3.83 | 3.75 |
| 0.50 | 0.41 | 0.42 | 0.44 | 0.46 | 0.45 | 0.57 | 0.57 |
| 2.55 | 2.23 | 2.27 | 2.37 | 2.50 | 2.53 | 2.87 | 2.82 |
| 0.41 | 0.28 | 0.29 | 0.30 | 0.31 | 0.32 | 0.40 | 0.39 |
| 0.65 | 0.52 | 1.64 | 0.52 | 0.52 | 0.32 | 0.61 | 0.58 |
| 0.49 | 0.32 | 0.32 | 0.34 | 0.35 | 0.34 | 0.46 | 0.45 |
| 0.26 | 0.14 | 0.14 | 0.11 | 0.10 | 0.10 | 0.15 | 0.13 |
|  |  |  |  |  |  |  |  |
| 1429 |  |  |  |  |  |  |  |
| 1.85 |  |  |  |  |  |  |  |
| 1412 |  |  |  |  |  |  |  |
| 1508 |  |  |  |  |  |  |  |
|  | 1390~1450 |  |  |  |  |  |  |

|  |  |  |  |  |  |  |
| --- | --- | --- | --- | --- | --- | --- |
| 17SCS00-37 | 17SCS00-41 | 17SCS00-45 | 17SCS00-47 |  |  |  |
| 75R-3-W94/98 | 79R-2-W139/143 | 81R-1-W109/112 | 83R-2-W61/65 | BCR-2 | BHVO-2 | AGV-2 |
| 1484.44 | 1502.88 | 1510.7 | 1526.09 |  |  |  |
| 1484.47 | 1502.91 | 1510.73 | 1526.12 |  |  |  |
|  |  |  |  |  |  |  |
| 49.79 | 48.35 | 49.84 | 49.76 | 54.8 | 50.08 |  |
| 1.602 | 1.539 | 1.526 | 1.468 | 2.318 | 2.773 |  |
| 16.11 | 16.13 | 16.71 | 16.65 | 13.22 | 13.43 |  |
| 8.73 | 11.16 | 8.84 | 9.11 | 14.05 | 12.45 |  |
| 7.857 | 10.044 | 7.956 | 8.199 | 12.645 | 11.205 |  |
| 0.167 | 0.143 | 0.209 | 0.183 | 0.199 | 0.172 |  |
| **6.93** | **5.86** | **6.78** | **6.88** | **3.61** | **7.18** |  |
| 11.37 | 11 | 11.32 | 11.16 | 7.22 | 11.47 |  |
| 2.87 | 3.19 | 3.17 | 3.1 | 3.32 | 2.43 |  |
| 0.43 | 0.24 | 0.08 | 0.07 | 1.82 | 0.52 |  |
| 0.17 | 0.208 | 0.179 | 0.18 | 0.363 | 0.28 |  |
| 1.8 | 2.99 | 2 | 1.6 | -0.2 | -0.57 |  |
| 99.98 | 100.8 | 100.65 | 100.17 | 100.72 | 100.21 |  |
|  |  |  |  |  |  |  |
| 31.0 | 17.8 | 16.1 | 12.8 | 10.1 | 4.80 | 11.1 |
| 0.40 | 0.69 | 0.44 | 0.53 | 2.17 | 1.08 | 2.19 |
| 39.8 | 36.3 | 37.2 | 36.2 | 33.3 | 31.6 | 13.4 |
| 267 | 263 | 261 | 250 | 415 | 320 | 118 |
| 225 | 212 | 240 | 257 | 15.5 | 283 | 15.0 |
| 50.9 | 33.3 | 51.0 | 41.7 | 37.0 | 44.9 | 16.3 |
| 102 | 96.9 | 124 | 132 | 12.2 | 121 | 18.4 |
| 87.5 | 68.6 | 83.4 | 85.3 | 20.5 | 128 | 53.0 |
| 77.4 | 83.4 | 78.7 | 76.0 | 132 | 103 | 86.8 |
| 16.6 | 17.1 | 16.8 | 16.4 | 21.9 | 21.2 | 20.7 |
| 1.57 | 1.78 | 1.56 | 1.56 | 1.81 | 1.64 | 1.19 |
| 9.06 | 2.92 | 0.93 | 0.78 | 46.3 | 9.89 | 66.0 |
| 157 | 167 | 173 | 171 | 338 | 395 | 665 |
| 35.3 | 35.5 | 32.8 | 31.9 | 35.6 | 25.9 | 20.2 |
| 112 | 114 | 112 | 107 | 185 | 169 | 234 |
| 5.74 | 5.88 | 5.73 | 5.36 | 12.5 | 18.5 | 14.3 |
| 0.14 | 0.059 | 0.029 | 0.023 | 1.11 | 0.11 | 1.14 |
| 17.4 | 29.0 | 23.1 | 22.2 | 675 | 130 | 1135 |
| 5.14 | 5.83 | 5.29 | 5.08 | 24.9 | 15.3 | 37.5 |
| 14.9 | 15.8 | 15.2 | 14.7 | 52.3 | 37.3 | 69.2 |
| 2.21 | 2.35 | 2.24 | 2.15 | 6.54 | 5.13 | 8.18 |
| 11.9 | 12.4 | 11.9 | 11.4 | 28.8 | 24.5 | 30.6 |
| 3.91 | 3.89 | 3.78 | 3.62 | 6.61 | 6.05 | 5.53 |
| 1.35 | 1.36 | 1.32 | 1.29 | 1.97 | 2.03 | 1.58 |
| 4.87 | 4.87 | 4.67 | 4.52 | 6.69 | 6.05 | 4.83 |
| 0.92 | 0.89 | 0.87 | 0.83 | 1.05 | 0.92 | 0.64 |
| 6.02 | 5.94 | 5.67 | 5.46 | 6.42 | 5.26 | 3.55 |
| 1.31 | 1.28 | 1.23 | 1.18 | 1.30 | 0.97 | 0.66 |
| 3.82 | 3.71 | 3.55 | 3.44 | 3.66 | 2.52 | 1.84 |
| 0.57 | 0.56 | 0.53 | 0.51 | 0.53 | 0.33 | 0.26 |
| 3.63 | 3.55 | 3.39 | 3.26 | 3.39 | 2.00 | 1.64 |
| 0.55 | 0.54 | 0.51 | 0.50 | 0.51 | 0.27 | 0.25 |
| 2.80 | 2.81 | 2.80 | 2.65 | 4.80 | 4.27 | 5.04 |
| 0.38 | 0.40 | 0.39 | 0.36 | 0.79 | 1.16 | 0.85 |
| 0.90 | 0.82 | 0.94 | 0.94 | 10.4 | 1.83 | 13.1 |
| 0.45 | 0.49 | 0.48 | 0.45 | 5.68 | 1.15 | 5.85 |
| 0.26 | 0.32 | 0.13 | 0.12 | 1.67 | 0.41 | 1.86 |
|  |  |  |  |  |  |  |
|  |  | 1364 | 1375 |  |  |  |
|  |  | 1.36 | 1.41 |  |  |  |
|  |  | 1325 | 1346 |  |  |  |
|  |  | 1394 | 1420 |  |  |  |
|  |  |  |  |  |  |  |

| **Table S2. Major elemental compositions of olivines from sample 67R-2-W68/71 and standard** | | | | | | |  |
| --- | --- | --- | --- | --- | --- | --- | --- |
| Sample name | No. | Na2O | SiO2 | Al2O3 | MgO | CaO | P2O5 |
| 67R-2-W68/71 | 1 | 0.005 | 39.1 | 0.046 | 39.5 | 0.309 | 0.164 |
| 67R-2-W68/71 | 2 | 0.043 | 39.4 | 0.032 | 42.4 | 0.307 | 0.074 |
| 67R-2-W68/71 | 3 | 0.049 | 40.4 | 0.015 | 44.3 | 0.233 | 0 |
| 67R-2-W68/71 | 4 | 0.085 | 40.8 | 0.8 | 41.3 | 1.634 | 0.033 |
| 67R-2-W68/71 | 5 | 0.042 | 40 | 0.041 | 43.7 | 0.289 | 0.115 |
| 67R-2-W68/71 | 6 | 0.026 | 40.2 | 0.043 | 43.7 | 0.325 | 0.123 |
| 67R-2-W68/71 | 7 | 0 | 39.6 | 0.036 | 43.3 | 0.328 | 0.049 |
| 67R-2-W68/71 | 8 | 0 | 39.3 | 0.061 | 40.5 | 0.37 | 0 |
| 67R-2-W68/71 | 9 | 0 | 39.2 | 0.02 | 41.2 | 0.313 | 0.148 |
| 67R-2-W68/71 | 10 | 0 | 39.8 | 0.068 | 43.2 | 0.271 | 0.09 |
| 67R-2-W68/71 | 11 | 0.028 | 39.7 | 0.014 | 43.2 | 0.261 | 0.066 |
| 67R-2-W68/71 | 12 | 0.023 | 40.2 | 0.057 | 43.4 | 0.34 | 0.082 |
| 67R-2-W68/71 | 13 | 0 | 39.2 | 0.107 | 42.1 | 0.305 | 0.09 |
| 67R-2-W68/71 | 14 | 0.053 | 38.8 | 0.028 | 40.4 | 0.37 | 0.114 |
| 67R-2-W68/71 | 15 | 0.046 | 37.3 | 0.1 | 38.8 | 0.357 | 0.154 |
| 67R-2-W68/71 | 16 | 0.007 | 39.1 | 0.051 | 41.2 | 0.293 | 0.18 |
| 67R-2-W68/71 | 17 | 0 | 38.8 | 0.033 | 42.8 | 0.285 | 0.107 |
| 67R-2-W68/71 | 18 | 0.012 | 39.2 | 0.047 | 42.2 | 0.241 | 0.205 |
| 67R-2-W68/71 | 19 | 0.019 | 38.8 | 0.087 | 39.5 | 0.389 | 0.041 |
| 67R-2-W68/71 | 20 | 0.022 | 38.5 | 0.054 | 40.2 | 0.368 | 0.041 |
| 67R-2-W68/71 | 21 | 0.027 | 38.6 | 0.074 | 39.5 | 0.355 | 0.09 |
| 67R-2-W68/71 | 22 | 0.04 | 38.3 | 0.145 | 41.1 | 0.347 | 0.065 |
| 67R-2-W68/71 | 23 | 0.014 | 39.8 | 0.047 | 43.8 | 0.275 | 0.171 |
| 67R-2-W68/71 | 24 | 0 | 39.6 | 0.078 | 43 | 0.265 | 0.179 |
| 67R-2-W68/71 | 25 | 0.023 | 39.4 | 0.067 | 42.6 | 0.223 | 0.269 |
| 67R-2-W68/71 | 26 | 0.031 | 39 | 0.065 | 42.6 | 0.286 | 0.139 |
| 67R-2-W68/71 | 27 | 0.007 | 39.2 | 0.018 | 42 | 0.298 | 0.081 |
| 67R-2-W68/71 | 28 | 0.017 | 39.3 | 0.045 | 41.9 | 0.303 | 0.106 |
| 67R-2-W68/71 | 29 | 0.021 | 39.2 | 0.027 | 41.5 | 0.278 | 0.033 |
| 67R-2-W68/71 | 30 | 0.029 | 39.4 | 0.016 | 41.5 | 0.283 | 0.057 |
| 67R-2-W68/71 | 31 | 0.007 | 38.9 | 0.042 | 43 | 0.401 | 0.253 |
| 67R-2-W68/71 | 32 | 0.07 | 39.7 | 0.053 | 43.9 | 0.335 | 0.229 |
| 67R-2-W68/71 | 33 | 0 | 39.8 | 0.015 | 44.6 | 0.299 | 0 |
| 67R-2-W68/71 | 34 | 0.016 | 39.9 | 0.063 | 43.8 | 0.317 | 0.066 |
| 67R-2-W68/71 | 35 | 0.021 | 40.5 | 0.022 | 43.7 | 0.384 | 0.049 |
| 67R-2-W68/71 | 36 | 0.023 | 40 | 0.034 | 43.6 | 0.322 | 0.106 |
| *standard* |  |  |  |  |  |  |  |
| SPI olivine ref. |  |  | 41.6 |  | 50.97 |  |  |
| SPI olivine | 1 | 0.002 | 41.4 | 0 | 50.7 | 0.035 | 0 |
| SPI olivine | 2 | 0 | 41.6 | 0.04 | 50.3 | 0.014 | 0.05 |
| SPI olivine | 3 | 0.011 | 41.4 | 0 | 50 | 0 | 0.05 |
| SPI olivine | 4 | 0.017 | 41.4 | 0 | 50 | 0 | 0 |
| SPI olivine | 5 | 0.015 | 41.8 | 0 | 51 | 0 | 0 |

|  |  |  |  |  |  |  |
| --- | --- | --- | --- | --- | --- | --- |
| NiO | CoO | FeO | MnO | Cr2O3 | TiO2 | Total |
| 0.116 | 0.102 | 20.6 | 0.336 | 0.121 | 0.116 | 100.5 |
| 0.109 | 0.07 | 17.8 | 0.314 | 0.071 | 0.123 | 100.7 |
| 0.142 | 0.047 | 15.2 | 0.254 | 0.076 | 0 | 100.6 |
| 0.206 | 0.01 | 15.5 | 0.267 | 0.043 | 0.261 | 101.1 |
| 0.206 | 0.054 | 15.3 | 0.286 | 0.037 | 0.01 | 100.1 |
| 0.152 | 0.009 | 15.3 | 0.234 | 0.178 | 0.023 | 100.3 |
| 0.163 | 0.082 | 15.8 | 0.246 | 0.082 | 0.057 | 99.8 |
| 0.136 | 0.08 | 18.6 | 0.308 | 0.048 | 0.099 | 99.6 |
| 0.102 | 0.033 | 17.8 | 0.285 | 0.083 | 0.013 | 99.2 |
| 0.22 | 0.059 | 16.3 | 0.317 | 0.088 | 0.033 | 100.5 |
| 0.161 | 0 | 16.1 | 0.253 | 0.102 | 0.03 | 100 |
| 0.163 | 0.024 | 16 | 0.255 | 0.112 | 0.02 | 100.6 |
| 0.068 | 0.057 | 16.7 | 0.319 | 0.23 | 0.007 | 99.2 |
| 0.143 | 0 | 19.8 | 0.302 | 0.062 | 0.02 | 100.1 |
| 0.094 | 0.066 | 18.1 | 0.335 | 3.474 | 0.069 | 98.8 |
| 0.107 | 0.04 | 19.1 | 0.311 | 0.024 | 0 | 100.4 |
| 0.129 | 0.03 | 17.5 | 0.297 | 0.028 | 0.053 | 100.1 |
| 0.13 | 0.078 | 17.7 | 0.292 | 0.077 | 0 | 100.2 |
| 0.114 | 0.057 | 19.8 | 0.314 | 0.042 | 0.089 | 99.3 |
| 0.107 | 0.073 | 19.9 | 0.302 | 0.018 | 0.026 | 99.6 |
| 0.082 | 0.059 | 19.9 | 0.307 | 0.091 | 0.02 | 99 |
| 0.165 | 0.06 | 16.9 | 0.272 | 0.117 | 0.03 | 97.6 |
| 0.142 | 0.069 | 16.5 | 0.269 | 0.168 | 0.02 | 101.4 |
| 0.172 | 0.073 | 16.6 | 0.269 | 0.077 | 0.043 | 100.3 |
| 0.169 | 0.028 | 16.5 | 0.257 | 0.067 | 0.03 | 99.6 |
| 0.215 | 0.012 | 17.1 | 0.264 | 0.071 | 0.046 | 99.9 |
| 0.099 | 0.04 | 18.1 | 0.292 | 0.024 | 0 | 100.2 |
| 0.142 | 0.062 | 18.1 | 0.313 | 0.076 | 0.069 | 100.4 |
| 0.147 | 0.071 | 18.1 | 0.327 | 0.048 | 0.007 | 99.7 |
| 0.131 | 0.047 | 18.2 | 0.306 | 0.046 | 0.016 | 100.1 |
| 0.169 | 0.076 | 16.1 | 0.271 | 0.107 | 0 | 99.3 |
| 0.165 | 0.012 | 15.6 | 0.236 | 0.175 | 0.023 | 100.6 |
| 0.214 | 0.045 | 15.3 | 0.279 | 0.081 | 0 | 100.6 |
| 0.196 | 0.002 | 15.3 | 0.242 | 0.035 | 0 | 99.9 |
| 0.242 | 0.049 | 15.5 | 0.249 | 0.089 | 0.01 | 100.8 |
| 0.153 | 0.083 | 16.6 | 0.261 | 0.128 | 0.06 | 101.4 |
|  |  |  |  |  |  |  |
| 0.37 |  | 7.25 |  |  |  |  |
| 0.315 | 0.042 | 7.2 | 0.076 | 0 | 0.078 | 99.8 |
| 0.41 | 0.039 | 7.1 | 0.099 | 0 | 0 | 99.7 |
| 0.304 | 0.035 | 7.2 | 0.115 | 0 | 0 | 99.1 |
| 0.371 | 0.09 | 7.3 | 0.083 | 0 | 0.007 | 99.2 |
| 0.399 | 0.007 | 7.3 | 0.155 | 0 | 0.007 | 100.7 |

|  |  |  |  |  |
| --- | --- | --- | --- | --- |
| Fo | Mn (ppm) | Ni (ppm) | Ca (ppm) | Fe/Mn |
| 77.5 | 2603 | 913 | 2207 | 61.3 |
| 81.1 | 2432 | 857 | 2193 | 56.6 |
| 84 | 1968 | 1117 | 1664 | 59.8 |
| 82.7 | 2068 | 1621 | 11671 | 58.2 |
| 83.7 | 2215 | 1621 | 2064 | 53.6 |
| 83.8 | 1813 | 1196 | 2321 | 65.2 |
| 83.2 | 1906 | 1282 | 2343 | 64.1 |
| 79.6 | 2386 | 1070 | 2643 | 60.5 |
| 80.7 | 2208 | 802 | 2236 | 62.5 |
| 82.6 | 2456 | 1731 | 1936 | 51.5 |
| 82.9 | 1960 | 1267 | 1864 | 63.6 |
| 83 | 1975 | 1282 | 2429 | 62.7 |
| 81.9 | 2471 | 535 | 2179 | 52.5 |
| 78.6 | 2339 | 1125 | 2643 | 65.7 |
| 79.5 | 2595 | 739 | 2550 | 53.9 |
| 79.6 | 2409 | 842 | 2093 | 61.3 |
| 81.4 | 2301 | 1015 | 2036 | 59.1 |
| 81.1 | 2262 | 1023 | 1721 | 60.6 |
| 78.2 | 2432 | 897 | 2779 | 63 |
| 78.4 | 2339 | 842 | 2629 | 65.9 |
| 78.1 | 2378 | 645 | 2536 | 64.7 |
| 81.4 | 2107 | 1298 | 2479 | 62.3 |
| 82.7 | 2084 | 1117 | 1964 | 61.4 |
| 82.4 | 2084 | 1353 | 1893 | 61.6 |
| 82.3 | 1991 | 1329 | 1593 | 64.2 |
| 81.7 | 2045 | 1691 | 2043 | 65 |
| 80.7 | 2262 | 779 | 2129 | 62 |
| 80.6 | 2425 | 1117 | 2164 | 57.9 |
| 80.5 | 2533 | 1156 | 1986 | 55.3 |
| 80.4 | 2370 | 1031 | 2021 | 59.6 |
| 82.8 | 2099 | 1329 | 2864 | 59.3 |
| 83.5 | 1828 | 1298 | 2393 | 66.3 |
| 84 | 2161 | 1683 | 2136 | 55 |
| 83.7 | 1875 | 1542 | 2264 | 63.4 |
| 83.5 | 1929 | 1904 | 2743 | 62.4 |
| 82.6 | 2022 | 1204 | 2300 | 63.5 |
|  |  |  |  |  |
|  |  |  |  |  |
|  |  |  |  |  |
|  |  |  |  |  |
|  |  |  |  |  |
|  |  |  |  |  |
|  |  |  |  |  |

| **Table S3. Major elemental compositions and calculated melting temperature and pressure for basalts with MgO>6.5% from Iceland** | | | | | | | | |  |  |  |  |  |  |  |  |  |  |  |
| --- | --- | --- | --- | --- | --- | --- | --- | --- | --- | --- | --- | --- | --- | --- | --- | --- | --- | --- | --- |
| SAMPLE ID | TECTONIC SETTING | SIO2 | TIO2 | AL2O3 | CR2O3 | FE2O3 | FE2O3T | FEO | FEOT | NIO | MNO | MGO | CAO | NA2O | K2O | P2O5 | T-melting | P-melting | REFERENCES |
| DAR0080-174-009 | SPREADING_CENTER | 49.52 | 1.37 | 14.35 |  | 13.46 | 13.46 |  |  |  | 0.2 | 7.46 | 11.63 | 2 | 0.13 | 0.14 | **1560** | **2.85** | Murton *et al.*^21^ |
| DAR0080-174-009 | SPREADING_CENTER | 49.79 | 1.617 | 13.19 |  | 1.53 | 16.55 | 13.52 |  |  | 0.19 | 6.48 | 11.1 | 2.06 | 0.19 | 0.16 | **1680** | **4.60** | Shorttle *et al.*^22^ |
| PETDB-3110-T1-1477 | SPREADING_CENTER | 49.26 | 1.705 | 14.185 |  | 13.715 | 13.72 |  |  |  | 0.205 | 6.95 | 12.02 | 1.76 | 0.19 | 0.14 | **1574** | **3.01** | Zellmer *et al.*^23^ |
| PETDB-3110-T2-1477 | SPREADING_CENTER | 49.65 | 1.565 | 14.73 |  | 12.805 | 12.81 |  |  |  | 0.195 | 7.02 | 12.48 | 1.935 | 0.165 | 0.13 | **1532** | **2.57** | Zellmer *et al.*^23^ |
| PETDB-3110-T5-871 AD | SPREADING_CENTER | 49.4 | 1.63 | 14.29 |  | 13.42 | 13.42 |  |  |  | 0.2 | 7.11 | 12.24 | 1.87 | 0.16 | 0.14 | **1560** | **2.87** | Zellmer *et al.*^23^ |
| PETDB-2222-HBT10 1 | INTRAPLATE_OFF-CRATON | 48.94 | 0.76 | 15.88 | 0.05 |  | 10.06 | 9.05 |  | 0.01 | 0.15 | 10.07 | 13.08 | 1.74 | 0.07 | 0.06 | **1417** | **1.59** | Thomson and Maclennan^24^ |
| PETDB-2222-HETA_CHIP1_GL1 | INTRAPLATE_OFF-CRATON | 48.82 | 0.75 | 15.88 | 0.06 |  | 10.49 | 9.44 |  |  | 0.16 | 9.52 | 13.26 | 1.74 | 0.09 | 0.06 | **1439** | **1.77** | Thomson and Maclennan^24^ |
| PETDB-2222-HBT10 2 | INTRAPLATE_OFF-CRATON | 48.25 | 0.74 | 15.29 | 0.03 |  | 11.26 | 10.13 |  | 0.01 | 0.15 | 10.02 | 12.98 | 1.74 | 0.06 | 0.05 | **1476** | **2.11** | Thomson and Maclennan^24^ |
| PETDB-2222-HET1_OL20_GL1 | INTRAPLATE_OFF-CRATON | 49.48 | 0.76 | 16.05 | 0.06 |  | 10.40 | 9.36 |  | 0.04 | 0.17 | 9.85 | 13.51 | 1.72 | 0.09 | 0.06 | **1427** | **1.67** | Thomson and Maclennan^24^ |
| PETDB-2222-HET1_OL25_GL1 | INTRAPLATE_OFF-CRATON | 49.49 | 0.78 | 16.25 | 0.06 |  | 10.99 | 9.89 |  | 0.02 | 0.16 | 9.76 | 13.32 | 1.78 | 0.07 | 0.06 | **1454** | **1.92** | Thomson and Maclennan^24^ |
| PETDB-2222-HET1_OL49_GL1 | INTRAPLATE_OFF-CRATON | 49.56 | 0.76 | 16.02 | 0.04 |  | 10.51 | 9.46 |  |  | 0.16 | 9.76 | 13.31 | 1.86 | 0.06 | 0.06 | **1433** | **1.72** | Thomson and Maclennan^24^ |
| END0025-008-SG15 | SPREADING_CENTER | 50.83 | 1.37 | 14.18 |  |  | 13.91 |  | 12.52 |  |  | 6.9 | 11.73 | 2.07 | 0.13 | 0.2 | **1569** | **2.86** | Melson *et al.*^25^ |
| RR00-070-005 | SPREADING_CENTER | 50.38 | 1.19 | 14.4 |  | 1.72 | 12.09 | 9.63 | 11.18 |  | 0.21 | 7.99 | 12.07 | 2.18 | 0.06 | 0.02 | **1495** | **2.14** | Bezos and Humler^26^ |
| RR00-097-006 | SPREADING_CENTER | 50.58 | 1.26 | 14.1 |  | 1.6 | 13.09 | 10.45 | 11.89 |  | 0.22 | 7.43 | 11.75 | 2.22 | 0.06 | 0.07 | **1539** | **2.56** | Bezos and Humler^26^ |
| RR00-145-001 | SPREADING_CENTER | 50.94 | 1.35 | 13.9 |  | 1.57 | 13.20 | 10.57 | 11.98 |  | 0.21 | 7.12 | 11.89 | 2.1 | 0.08 | 0.09 | **1542** | **2.53** | Bezos and Humler^26^ |
| END0025-008-001 | SPREADING_CENTER | 51.05 | 1.38 | 14.02 |  |  | 13.37 | 11.23 | 12.49 |  |  | 6.79 | 11.76 | 2.07 | 0.15 | 0.15 | **1547** | **2.56** | Melson *et al*.^25^, Christie *et al*.^27^ |
| END0025-008-002 | SPREADING_CENTER | 50.59 | 1.43 | 13.95 |  |  | 14.01 |  | 12.61 |  |  | 6.67 | 11.7 | 2.05 | 0.14 | 0.16 | **1575** | **2.93** | Melson *et al.*^25^ |
| END0025-008-003 | SPREADING_CENTER | 50.96 | 1.44 | 13.72 |  |  | 14.33 |  | 12.9 |  |  | 6.69 | 11.68 | 2.06 | 0.14 | 0.15 | **1585** | **3.03** | Melson *et al.*^25^ |
| END0025-008-004 | SPREADING_CENTER | 50.55 | 1.38 | 14.07 |  |  | 13.64 |  | 12.28 |  |  | 6.82 | 11.72 | 2.04 | 0.14 | 0.14 | **1562** | **2.77** | Melson *et al*.^25^, Blichert-Toft *et al*.^28^, Kelly *et al*.^29^ |
| END0025-008-SG02 | SPREADING_CENTER | 50.53 | 1.38 | 13.94 |  |  | 13.78 |  | 12.4 |  |  | 6.78 | 11.68 | 2.05 | 0.11 | 0.14 | **1567** | **2.81** | Melson *et al.*^25^ |
| END0025-008-SG03 | SPREADING_CENTER | 50.71 | 1.39 | 13.9 |  |  | 13.88 |  | 12.49 |  |  | 6.79 | 11.69 | 2.04 | 0.12 | 0.17 | **1569** | **2.83** | Melson *et al.*^25^ |
| END0025-008-SG04 | SPREADING_CENTER | 51.04 | 1.42 | 13.92 |  |  | 13.79 |  | 12.41 |  |  | 6.73 | 11.78 | 2.06 | 0.12 | 0.16 | **1563** | **2.75** | Melson *et al.*^25^ |
| END0025-008-SG05 | SPREADING_CENTER | 50.75 | 1.42 | 13.98 |  |  | 13.76 |  | 12.38 |  |  | 6.9 | 11.72 | 2.03 | 0.13 | 0.16 | **1563** | **2.76** | Melson *et al.*^25^ |
| END0025-008-SG06 | SPREADING_CENTER | 50.84 | 1.48 | 13.8 |  |  | 14.12 |  | 12.71 |  |  | 6.62 | 11.69 | 2.07 | 0.13 | 0.15 | **1579** | **2.95** | Melson *et al.*^25^ |
| END0025-008-SG07 | SPREADING_CENTER | 50.97 | 1.42 | 13.83 |  |  | 13.86 |  | 12.47 |  |  | 6.68 | 11.73 | 2.06 | 0.13 | 0.14 | **1568** | **2.80** | Melson *et al.*^25^ |
| END0025-008-SG08 | SPREADING_CENTER | 51.22 | 1.43 | 13.73 |  |  | 13.90 |  | 12.51 |  |  | 6.74 | 11.74 | 2.06 | 0.13 | 0.16 | **1565** | **2.75** | Melson *et al.*^25^ |
| END0025-008-SG09 | SPREADING_CENTER | 51.04 | 1.44 | 14.01 |  |  | 13.80 |  | 12.42 |  |  | 6.79 | 11.53 | 2 | 0.12 | 0.18 | **1564** | **2.73** | Melson *et al.*^25^ |
| END0025-008-SG10 | SPREADING_CENTER | 51.15 | 1.44 | 14.16 |  |  | 14.01 |  | 12.61 |  |  | 6.52 | 11.66 | 2.02 | 0.12 | 0.16 | **1571** | **2.84** | Melson *et al.*^25^ |
| END0025-008-SG12 | SPREADING_CENTER | 51.21 | 1.41 | 14.01 |  |  | 13.81 |  | 12.43 |  |  | 6.76 | 11.75 | 2.04 | 0.13 | 0.17 | **1563** | **2.74** | Melson *et al.*^25^ |
| END0025-008-SG13 | SPREADING_CENTER | 50.66 | 1.42 | 14.09 |  |  | 13.78 |  | 12.4 |  |  | 6.71 | 11.67 | 2.01 | 0.13 | 0.18 | **1567** | **2.81** | Melson *et al.*^25^ |
| END0025-008-SG14 | SPREADING_CENTER | 50.79 | 1.41 | 14.01 |  |  | 13.90 |  | 12.51 |  |  | 6.67 | 11.7 | 2.05 | 0.11 | 0.17 | **1569** | **2.82** | Melson *et al.*^25^ |
| END0025-009-SG01 | SPREADING_CENTER | 50.86 | 1.5 | 14.05 |  |  | 13.78 |  | 12.4 |  |  | 6.66 | 11.78 | 2.09 | 0.19 | 0.16 | **1563** | **2.79** | Melson *et al.*^25^ |
| END0025-009-SG02 | SPREADING_CENTER | 50.51 | 1.49 | 13.95 |  |  | 13.63 |  | 12.27 |  |  | 6.86 | 11.81 | 2.08 | 0.16 | 0.15 | **1561** | **2.78** | Melson *et al.*^25^ |
| END0025-009-SG03 | SPREADING_CENTER | 50.92 | 1.5 | 14.05 |  |  | 13.82 |  | 12.44 |  |  | 6.78 | 11.86 | 2.11 | 0.17 | 0.17 | **1566** | **2.84** | Melson *et al.*^25^ |
| END0025-009-SG04 | SPREADING_CENTER | 50.81 | 1.54 | 13.96 |  |  | 13.66 |  | 12.29 |  |  | 6.66 | 11.87 | 2.11 | 0.16 | 0.18 | **1561** | **2.76** | Melson *et al.*^25^ |
| END0025-009-SG05 | SPREADING_CENTER | 50.92 | 1.51 | 13.94 |  |  | 13.66 |  | 12.29 |  |  | 6.68 | 11.69 | 2.04 | 0.17 | 0.17 | **1559** | **2.71** | Melson *et al.*^25^ |
| END0025-009-SG06 | SPREADING_CENTER | 50.54 | 1.55 | 14.1 |  |  | 13.47 |  | 12.12 |  |  | 6.77 | 11.86 | 2.1 | 0.17 | 0.17 | **1556** | **2.73** | Melson *et al.*^25^ |
| END0025-009-SG07 | SPREADING_CENTER | 50.32 | 1.52 | 13.87 |  |  | 13.69 |  | 12.32 |  |  | 6.7 | 11.9 | 2.08 | 0.16 | 0.17 | **1567** | **2.86** | Melson *et al.*^25^ |
| END0025-009-SG08 | SPREADING_CENTER | 50.79 | 1.5 | 13.8 |  |  | 13.71 |  | 12.34 |  |  | 6.82 | 11.72 | 2.09 | 0.16 | 0.18 | **1561** | **2.75** | Melson *et al.*^25^ |
| END0025-009-SG09 | SPREADING_CENTER | 50.63 | 1.5 | 13.77 |  |  | 14.00 |  | 12.6 |  |  | 6.62 | 11.77 | 2.08 | 0.17 | 0.18 | **1577** | **2.96** | Melson *et al.*^25^ |
| END0025-009-SG10 | SPREADING_CENTER | 50.52 | 1.54 | 13.85 |  |  | 13.68 |  | 12.31 |  |  | 6.73 | 11.78 | 2.07 | 0.17 | 0.19 | **1563** | **2.79** | Melson *et al.*^25^ |
| END0025-008-SG16 | SPREADING_CENTER | 50.58 | 1.42 | 14.06 |  |  | 13.86 |  | 12.47 |  |  | 6.76 | 11.71 | 2.06 | 0.15 | 0.17 | **1569** | **2.86** | Melson *et al.*^25^ |
| END0025-008-SG17 | SPREADING_CENTER | 50.57 | 1.43 | 13.91 |  |  | 14.01 |  | 12.61 |  |  | 6.54 | 11.64 | 2.1 | 0.13 | 0.18 | **1578** | **2.96** | Melson *et al.*^25^ |
| END0025-009-001 | SPREADING_CENTER | 50.28 | 1.37 | 14.38 |  |  | 13.17 |  | 11.85 |  |  | 7.09 | 11.77 | 2.06 | 0.15 | 0.16 | **1543** | **2.60** | Melson *et al*.^25^, Blichert-Toft *et al*.^28^, Kelly *et al*.^29^ |
| END0025-009-002 | SPREADING_CENTER | 50.74 | 1.43 | 14.69 |  |  | 13.24 |  | 11.92 |  |  | 7.14 | 11.89 | 2.07 | 0.16 | 0.15 | **1540** | **2.57** | Melson *et al.*^25^ |
| END0025-009-003 | SPREADING_CENTER | 50.06 | 1.4 | 14.46 |  |  | 13.17 |  | 11.85 |  |  | 7.11 | 11.77 | 2.07 | 0.16 | 0.15 | **1545** | **2.65** | Melson *et al.*^25^ |
| END0025-009-004 | SPREADING_CENTER | 50.13 | 1.37 | 14.46 |  |  | 13.06 |  | 11.75 |  |  | 7.34 | 11.77 | 2.02 | 0.16 | 0.17 | **1538** | **2.55** | Melson *et al.*^25^ |
| *BA52-005-001 | SPREADING_CENTER | 50.82 | 1.59 | 14.21 |  |  | 14.28 |  | 12.85 |  |  | 6.61 | 11.25 | 2.16 | 0.16 | 0.15 | **1583** | **3.05** | Melson *et al.*^25^ |
| *BA52-005-033 | SPREADING_CENTER | 50.57 | 1.64 | 13.96 |  |  | 13.68 |  | 12.31 |  |  | 7.05 | 11.26 | 2.15 | 0.17 | 0.19 | **1561** | **2.77** | Melson *et al.*^25^ |
| *BA52-005-034 | SPREADING_CENTER | 50.27 | 1.64 | 14.14 |  |  | 13.74 |  | 12.37 |  |  | 6.65 | 11.06 | 2.25 | 0.19 | 0.19 | **1566** | **2.87** | Melson *et al.*^25^ |
| *BA52-005-035 | SPREADING_CENTER | 50.56 | 1.63 | 14.34 |  |  | 13.72 |  | 12.35 |  |  | 6.6 | 11.23 | 2.14 | 0.18 | 0.16 | **1564** | **2.82** | Melson *et al.*^25^ |
| *BA52-005-036 | SPREADING_CENTER | 50.26 | 1.62 | 14.28 |  |  | 13.64 |  | 12.28 |  |  | 6.54 | 11.11 | 2.18 | 0.14 | 0.18 | **1563** | **2.81** | Melson *et al.*^25^ |
| *BA52-005-037 | SPREADING_CENTER | 50.11 | 1.62 | 14.14 |  |  | 13.71 |  | 12.34 |  |  | 6.49 | 11.31 | 2.18 | 0.19 | 0.18 | **1569** | **2.91** | Melson *et al.*^25^ |
| *BA52-005-038 | SPREADING_CENTER | 50.9 | 1.61 | 14.18 |  |  | 13.79 |  | 12.41 |  |  | 6.57 | 11.32 | 2.17 | 0.18 | 0.17 | **1565** | **2.81** | Melson *et al.*^25^ |
| *BA52-005-039 | SPREADING_CENTER | 50.27 | 1.52 | 14 |  |  | 13.77 |  | 12.39 |  |  | 6.55 | 11.28 | 2.2 | 0.17 | 0.16 | **1570** | **2.89** | Melson *et al.*^25^ |
| *BA52-005-040 | SPREADING_CENTER | 50.95 | 1.55 | 13.81 |  |  | 13.88 |  | 12.49 |  |  | 6.72 | 11.25 | 2.15 | 0.17 | 0.16 | **1568** | **2.81** | Melson *et al.*^25^ |
| *BA52-005-041 | SPREADING_CENTER | 50.19 | 1.45 | 14.27 |  |  | 13.57 |  | 12.21 |  |  | 6.68 | 11.45 | 2.15 | 0.16 | 0.19 | **1563** | **2.82** | Melson *et al.*^25^ |
| *BA52-005-043 | SPREADING_CENTER | 50.61 | 1.49 | 13.85 |  |  | 13.73 |  | 12.36 |  |  | 6.79 | 11.58 | 2.15 | 0.18 | 0.19 | **1564** | **2.80** | Melson *et al.*^25^ |
| *BA52-005-044 | SPREADING_CENTER | 50.89 | 1.6 | 14.18 |  |  | 13.86 |  | 12.47 |  |  | 6.65 | 11.51 | 2.12 | 0.21 | 0.18 | **1568** | **2.86** | Melson *et al.*^25^ |
| *BA52-005-045 | SPREADING_CENTER | 50.55 | 1.54 | 14.03 |  |  | 13.88 |  | 12.49 |  |  | 6.77 | 11.57 | 2.17 | 0.2 | 0.18 | **1570** | **2.91** | Melson *et al.*^25^ |
| *BA52-005-046 | SPREADING_CENTER | 50.78 | 1.64 | 14.04 |  |  | 13.76 |  | 12.38 |  |  | 6.88 | 11.56 | 2.21 | 0.17 | 0.18 | **1563** | **2.82** | Melson *et al.*^25^ |
| *BA52-005-047 | SPREADING_CENTER | 50.09 | 1.56 | 13.85 |  |  | 13.80 |  | 12.42 |  |  | 6.7 | 11.42 | 2.21 | 0.17 | 0.2 | **1573** | **2.95** | Melson *et al.*^25^ |
| *BA52-005-048 | SPREADING_CENTER | 50.55 | 1.58 | 14.03 |  |  | 13.59 |  | 12.23 |  |  | 6.66 | 11.44 | 2.17 | 0.21 | 0.24 | **1560** | **2.78** | Melson *et al.*^25^ |
| *BA52-005-049 | SPREADING_CENTER | 49.41 | 1.54 | 14.39 |  |  | 13.67 |  | 12.3 |  |  | 6.65 | 11.28 | 2.09 | 0.17 | 0.15 | **1571** | **2.96** | Melson *et al.*^25^ |
| *BA52-005-050 | SPREADING_CENTER | 49.94 | 1.46 | 14.22 |  |  | 13.36 |  | 12.02 |  |  | 6.48 | 11.26 | 2.11 | 0.16 | 0.21 | **1557** | **2.74** | Melson *et al.*^25^ |
| *BA52-005-051 | SPREADING_CENTER | 50.64 | 1.59 | 14.68 |  |  | 13.87 |  | 12.48 |  |  | 6.84 | 11.41 | 2.14 | 0.17 | 0.16 | **1565** | **2.87** | Jenner and O'Neill^30^, Reekie *et al*.^31^ |
| *BA52-005-052 | SPREADING_CENTER | 50.74 | 1.58 | 14.54 |  |  | 13.73 |  | 12.36 |  |  | 7.02 | 11.36 | 2.1 | 0.15 | 0.22 | **1559** | **2.78** | Jenner and O'Neill^30^, Reekie *et al*.^31^ |
| *BA52-005-053 | SPREADING_CENTER | 50.42 | 1.52 | 14.36 |  |  | 13.82 |  | 12.44 |  |  | 6.64 | 11.31 | 2.18 | 0.17 | 0.16 | **1569** | **2.90** | Jenner and O'Neill^30^, Reekie *et al*.^31^ |
| *BA52-005-054 | SPREADING_CENTER | 50.32 | 1.53 | 14.46 |  |  | 13.86 |  | 12.47 |  |  | 6.76 | 11.37 | 2.12 | 0.19 | 0.19 | **1570** | **2.93** | Jenner and O'Neill^30^, Reekie *et al*.^31^ |
| *BA52-005-055 | SPREADING_CENTER | 50.84 | 1.48 | 14.27 |  |  | 13.68 |  | 12.31 |  |  | 7.18 | 11.33 | 2.17 | 0.16 | 0.2 | **1557** | **2.74** | Jenner and O'Neill^30^, Reekie *et al*.^31^ |
| *BA52-005-056 | SPREADING_CENTER | 50.54 | 1.58 | 14.38 |  |  | 13.51 |  | 12.16 |  |  | 6.94 | 11.52 | 2.11 | 0.18 | 0.16 | **1553** | **2.71** | Jenner and O'Neill^30^, Reekie *et al*.^31^ |
| *BA52-005-057 | SPREADING_CENTER | 51.39 | 1.56 | 14.5 |  |  | 13.62 |  | 12.26 |  |  | 7.07 | 11.36 | 2.11 | 0.18 | 0.12 | **1548** | **2.61** | Jenner and O'Neill^30^, Reekie *et al*.^31^ |
| *BA52-005-058 | SPREADING_CENTER | 50.66 | 1.52 | 14.52 |  |  | 13.80 |  | 12.42 |  |  | 6.83 | 11.38 | 2.14 | 0.18 | 0.15 | **1564** | **2.84** | Jenner and O'Neill^30^, Reekie *et al*.^31^ |
| *BA52-005-059 | SPREADING_CENTER | 50.93 | 1.54 | 14.16 |  |  | 14.01 |  | 12.61 |  |  | 6.61 | 11.27 | 2.13 | 0.18 | 0.16 | **1571** | **2.87** | Jenner and O'Neill^30^, Reekie *et al*.^31^ |
| *BA52-005-060 | SPREADING_CENTER | 51.22 | 1.53 | 14.07 |  |  | 13.69 |  | 12.32 |  |  | 6.68 | 11.42 | 2.12 | 0.18 | 0.15 | **1557** | **2.68** | Jenner and O'Neill^30^, Reekie *et al*.^31^ |
| *BA52-005-061 | SPREADING_CENTER | 51.13 | 1.52 | 14.54 |  |  | 14.00 |  | 12.6 |  |  | 6.58 | 11.54 | 2.1 | 0.16 | 0.12 | **1568** | **2.85** | Melson *et al.*^25^ |
| *BA52-005-062 | SPREADING_CENTER | 49.63 | 1.53 | 13.98 |  |  | 13.93 |  | 12.54 |  |  | 6.75 | 11.45 | 2.08 | 0.16 | 0.08 | **1582** | **3.08** | Melson *et al.*^25^ |
| *BA52-005-064 | SPREADING_CENTER | 50.65 | 1.55 | 14.21 |  |  | 13.94 |  | 12.55 |  |  | 7.47 | 11.36 | 2.04 | 0.16 | 0.1 | **1568** | **2.87** | Melson *et al.*^25^ |
| *BA52-005-065 | SPREADING_CENTER | 50.25 | 1.5 | 13.75 |  |  | 14.08 |  | 12.67 |  |  | 6.93 | 11.41 | 2.07 | 0.15 | 0.11 | **1580** | **3.00** | Melson *et al.*^25^ |
| *BA52-005-066 | SPREADING_CENTER | 50.63 | 1.66 | 14.03 |  |  | 14.22 |  | 12.8 |  |  | 6.56 | 11.39 | 2.13 | 0.19 | 0.12 | **1583** | **3.06** | Melson *et al.*^25^ |
| *BA52-005-067 | SPREADING_CENTER | 50.55 | 1.56 | 14.02 |  |  | 14.21 |  | 12.79 |  |  | 6.85 | 11.48 | 2.18 | 0.2 | 0.12 | **1583** | **3.09** | Melson *et al.*^25^ |
| *BA52-005-VG1879 | SPREADING_CENTER | 50.07 | 1.63 | 13.75 |  |  | 13.74 |  | 12.37 |  |  | 6.84 | 11.39 | 2.05 | 0.17 | 0.16 | **1570** | **2.87** | Melson *et al.*^25^ |
| *BA52-005-VG2329 | SPREADING_CENTER | 50.41 | 1.61 | 13.58 |  |  | 14.39 |  | 12.95 |  |  | 6.59 | 11.32 | 2.16 | 0.2 | 0.13 | **1593** | **3.18** | Melson *et al.*^25^ |
| *BA52-005-002-VG2201 | SPREADING_CENTER | 49.85 | 1.54 | 14.07 |  |  | 13.60 |  | 12.24 |  |  | 6.95 | 11.31 | 2.17 | 0.15 | 0.14 | **1565** | **2.87** | Melson *et al.*^25^ |
| *BA52-005-002-VG2202 | SPREADING_CENTER | 50.87 | 1.52 | 14.38 |  |  | 13.76 |  | 12.38 |  |  | 6.69 | 11.28 | 2.11 | 0.17 | 0.15 | **1562** | **2.77** | Melson *et al.*^25^ |
| *BA52-005-004 | SPREADING_CENTER | 50.31 | 1.52 | 14.13 |  |  | 13.59 |  | 12.23 |  |  | 7.26 | 11.25 | 2.14 | 0.16 | 0.18 | **1558** | **2.77** | Melson *et al.*^25^ |
| *BA52-005-005 | SPREADING_CENTER | 50.38 | 1.54 | 14.46 |  |  | 13.58 |  | 12.22 |  |  | 6.91 | 11.29 | 2.1 | 0.17 | 0.18 | **1558** | **2.76** | Melson *et al.*^25^ |
| *BA52-005-006 | SPREADING_CENTER | 50.93 | 1.6 | 14.02 |  |  | 13.99 |  | 12.59 |  |  | 6.63 | 11.35 | 2.13 | 0.18 | 0.13 | **1572** | **2.88** | Melson *et al.*^25^ |
| *BA52-005-007 | SPREADING_CENTER | 50.74 | 1.58 | 14.29 |  |  | 13.77 |  | 12.39 |  |  | 7.07 | 11.33 | 2.1 | 0.18 | 0.15 | **1560** | **2.78** | Melson *et al.*^25^ |
| *BA52-005-008 | SPREADING_CENTER | 50.99 | 1.57 | 14.8 |  |  | 13.64 |  | 12.28 |  |  | 7.14 | 11.39 | 2.05 | 0.2 | 0.18 | **1552** | **2.70** | Melson *et al.*^25^ |
| *BA52-005-009 | SPREADING_CENTER | 51.13 | 1.58 | 14.24 |  |  | 13.63 |  | 12.27 |  |  | 6.94 | 11.26 | 2.1 | 0.19 | 0.16 | **1555** | **2.67** | Melson *et al.*^25^ |
| *BA52-005-010 | SPREADING_CENTER | 50.7 | 1.64 | 14.14 |  |  | 13.94 |  | 12.55 |  |  | 6.61 | 11.42 | 2.1 | 0.18 | 0.18 | **1573** | **2.91** | Melson *et al.*^25^ |
| *BA52-005-011 | SPREADING_CENTER | 50.73 | 1.56 | 13.95 |  |  | 13.72 |  | 12.35 |  |  | 6.57 | 11.38 | 2.25 | 0.17 | 0.11 | **1563** | **2.80** | Melson *et al.*^25^ |
| *BA52-005-012 | SPREADING_CENTER | 51 | 1.56 | 13.86 |  |  | 13.77 |  | 12.39 |  |  | 7.11 | 11.43 | 2.18 | 0.21 | 0.12 | **1561** | **2.77** | Melson *et al.*^25^ |
| *BA52-005-013 | SPREADING_CENTER | 50.34 | 1.48 | 13.96 |  |  | 13.81 |  | 12.43 |  |  | 6.96 | 11.23 | 2.03 | 0.17 | 0.14 | **1567** | **2.83** | Melson *et al.*^25^ |
| *BA52-005-014 | SPREADING_CENTER | 50.92 | 1.64 | 14.09 |  |  | 14.00 |  | 12.6 |  |  | 6.51 | 11.35 | 2.17 | 0.2 | 0.14 | **1572** | **2.90** | Melson *et al.*^25^ |
| *BA52-005-015 | SPREADING_CENTER | 50.56 | 1.58 | 14.13 |  |  | 13.77 |  | 12.39 |  |  | 6.95 | 11.42 | 2.18 | 0.18 | 0.12 | **1565** | **2.85** | Melson *et al.*^25^ |
| *BA52-005-016 | SPREADING_CENTER | 50.11 | 1.46 | 14.38 |  |  | 13.79 |  | 12.41 |  |  | 6.78 | 11.34 | 2.1 | 0.16 | 0.15 | **1571** | **2.93** | Melson *et al.*^25^ |
| *BA52-005-017 | SPREADING_CENTER | 51.22 | 1.61 | 14.08 |  |  | 13.71 |  | 12.34 |  |  | 7.04 | 11.36 | 2.15 | 0.17 | 0.13 | **1558** | **2.71** | Melson *et al.*^25^ |
| *BA52-005-018 | SPREADING_CENTER | 50.44 | 1.45 | 13.88 |  |  | 13.79 |  | 12.41 |  |  | 7.16 | 11.39 | 2.12 | 0.19 | 0.15 | **1567** | **2.86** | Melson *et al.*^25^ |
| *BA52-005-019 | SPREADING_CENTER | 50.89 | 1.5 | 14.21 |  |  | 13.72 |  | 12.35 |  |  | 7.08 | 11.5 | 2.25 | 0.16 | 0.14 | **1558** | **2.78** | Melson *et al.*^25^ |
| *BA52-005-020 | SPREADING_CENTER | 51.18 | 1.5 | 14.2 |  |  | 13.77 |  | 12.39 |  |  | 7.15 | 11.57 | 2.16 | 0.15 | 0.13 | **1558** | **2.74** | Melson *et al.*^25^ |
| *BA52-005-021 | SPREADING_CENTER | 51.09 | 1.5 | 14.55 |  |  | 13.56 |  | 12.2 |  |  | 6.74 | 11.22 | 2.1 | 0.2 | 0.16 | **1553** | **2.66** | Melson *et al.*^25^ |
| *BA52-005-022 | SPREADING_CENTER | 51.38 | 1.67 | 13.97 |  |  | 14.30 |  | 12.87 |  |  | 6.64 | 11.15 | 2.14 | 0.2 | 0.2 | **1578** | **2.93** | Melson *et al.*^25^ |
| *BA52-005-023 | SPREADING_CENTER | 51.43 | 1.61 | 14.17 |  |  | 14.17 |  | 12.75 |  |  | 6.72 | 11.32 | 2.1 | 0.21 | 0.18 | **1572** | **2.87** | Melson *et al.*^25^ |
| *BA52-005-024 | SPREADING_CENTER | 50.55 | 1.57 | 14.55 |  |  | 13.70 |  | 12.33 |  |  | 6.64 | 11.17 | 2.1 | 0.16 | 0.2 | **1562** | **2.78** | Melson *et al.*^25^ |
| *BA52-005-025 | SPREADING_CENTER | 50.55 | 1.54 | 14.2 |  |  | 13.67 |  | 12.3 |  |  | 6.79 | 11.26 | 2.11 | 0.18 | 0.17 | **1560** | **2.75** | Melson *et al.*^25^ |
| *BA52-005-026 | SPREADING_CENTER | 51.39 | 1.57 | 14.29 |  |  | 13.76 |  | 12.38 |  |  | 6.97 | 11.33 | 2.1 | 0.17 | 0.17 | **1555** | **2.66** | Melson *et al.*^25^ |
| *BA52-005-027 | SPREADING_CENTER | 51.23 | 1.59 | 14.12 |  |  | 13.78 |  | 12.4 |  |  | 6.58 | 11.34 | 2.13 | 0.16 | 0.17 | **1560** | **2.72** | Melson *et al.*^25^ |
| *BA52-005-028 | SPREADING_CENTER | 51.39 | 1.6 | 14.19 |  |  | 13.80 |  | 12.42 |  |  | 7.06 | 11.39 | 2.13 | 0.2 | 0.17 | **1557** | **2.71** | Melson *et al.*^25^ |
| *BA52-005-029 | SPREADING_CENTER | 51.3 | 1.58 | 14.14 |  |  | 13.64 |  | 12.28 |  |  | 6.81 | 11.36 | 2.06 | 0.18 | 0.16 | **1554** | **2.63** | Melson *et al.*^25^ |
| *BA52-005-030-VG2229 | SPREADING_CENTER | 51.29 | 1.52 | 14.39 |  |  | 13.74 |  | 12.37 |  |  | 7.22 | 11.45 | 2.08 | 0.16 | 0.17 | **1556** | **2.70** | Melson *et al.*^25^ |
| *BA52-005-030-VG2230 | SPREADING_CENTER | 50.77 | 1.61 | 14.32 |  |  | 13.66 |  | 12.29 |  |  | 7.05 | 11.13 | 2.17 | 0.17 | 0.17 | **1556** | **2.73** | Melson *et al.*^25^ |
| *BA52-005-030-VG2231 | SPREADING_CENTER | 50.85 | 1.61 | 14.26 |  |  | 13.79 |  | 12.41 |  |  | 6.82 | 11.19 | 2.15 | 0.17 | 0.17 | **1562** | **2.78** | Melson *et al.*^25^ |
| Data for basalts from Iceland are downloaded from Petrological Database (http://www.earthchem.org/petdb). | | | | | |  |  |  |  |  |  |  |  |  |  |  | 1558 | 2.77 |  |

**Supplementary References**

1. Jochum, K. P., Weis, U., Schwager, B., Stoll, B. & Enzweiler, J. Reference values following ISO guidelines for frequently requested rock reference materials. *Geostand. Geoanal. Res.* **40**, 333-350 (2016).
2. Cheng, H., Liu, X. C., Vervoort, J. D., Wilford, D. & Cao, D. D. Micro-sampling Lu–Hf geochronology reveals episodic garnet growth and multiple high-pressure metamorphic events. *J. Metamorph. Geol.* **34**, 363-377 (2016).
3. Lee, C. T. A., Luffi, P., Plank, T., Dalton, H. & Leeman, W. P. Constraints on the depths and temperatures of basaltic magma generation on Earth and other terrestrial planets using new thermobarometers for mafic magmas. *Earth Planet. Sci. Lett.* **279**, 20-33 (2009).
4. Herzberg, C., & O’Hara, M. J. Plume-associated ultramafic magmas of Phanerozoic age. *J. Petrol.* **43**, 1857-1883 (2002).
5. Herzberg, C. & Asimow, P. D. PRIMELT3 MEGA.XLSM software for primary magma calculation: Peridotite primary magma MgO contents from the liquidus to the solidus. *Geochem. Geophys. Geosyst.* **16**, 563-578 (2015).
6. Walter, M. J. Melting of garnet peridotite and the origin of komatiite and depleted lithosphere. *J. Petrol.* **39**, 29-60 (1998).
7. Toplis, M. J. The thermodynamics of iron and magnesium partitioning between olivine and liquid: Criteria for assessing and predicting equilibrium in natural and experimental systems. *Contrib. Mineral. Petrol.* **149**, 22-30 (2005).
8. Putirka, K. D. Mantle potential temperatures at Hawaii, Iceland, and the mid-ocean ridge system, as inferred from olivine phenocrysts: Evidence for thermally driven mantle plumes. *Geochem. Geophys. Geosyst.* **6**, Q05L08 (2005).
9. Roeder, P. L., & Emslie, R. F. Olivine-liquid equilibrium. *Contrib. Mineral. Petrol.* **29**, 275-289 (1970).
10. Langmuir, C. H., & Hanson, G. H. Calculating mineral-melt equilibria with stoichiometry, mass balance, and single component distribution coefficients in *Thermodynamics of Minerals and Melts* (ed. Newton, R. C., Navrotsky, A., & Wood, B. J.) 247-271 (Springer, 1981).
11. Zhang, G.-L., Sun, W.-D. & Seward, G. Mantle source and magmatic evolution of the dying spreading ridge in the South China Sea. *Geochem. Geophys. Geosyst.* **19**, 4385-4399 (2018).
12. Zhang, G.-L. *et al*. Geochemical nature of sub-ridge mantle and opening dynamics of the South China Sea. *Earth Planet. Sci. Lett.* **489**, 145-155 (2018).
13. McDonough, W. F. & Sun, S. S. The composition of the Earth. *Chem. Geol.* **120**, 223-253 (1995).
14. Gale, A., Dalton, C. A., Langmuir, C. H., Su, Y. & Schilling, J. G. The mean composition of ocean ridge basalts. *Geochem. Geophys. Geosyst.* **14**, 489-518 (2013).
15. Herzberg, C. Identification of source lithology in the Hawaiian and Canary Islands: Implications for origins. *J. Petrol.* **52**, 113-146 (2011).
16. Sobolev, A. V. *et al*. The amount of recycled crust in sources of mantle-derived melts. *Science* **316**, 412-417 (2007).
17. Liu, J.-Q. *et al*. Petrogenesis of late Cenozoic basalts from North Hainan Island: Constraints from melt inclusions and their host olivines. *Geochim. Cosmochim. Acta* **152**, 89-121 (2015).
18. Sato, H. Nickel content of basaltic magmas: Identification of primary magmas and a measure of the degree of olivine fraction. *Lithos* **10**, 113-120 (1977).
19. Johnson, K. T. M., Dick, H. J. B., & Shimizu, N. Melting in the oceanic upper mantle: An ion microprobe study of diopsides in abyssal peridotites. *J. Geophys. Res.* **95**, 2661-2678 (1990).
20. Workman, R. K., & Hart, S. R. Major and trace element composition of the depleted MORB mantle (DMM). *Earth Planet. Sci. Lett.* **231**, 53-72 (2005).
21. Murton, B. J., Taylor, R. N., & Thirlwall, M. F. Plume-Ridge interaction: a geochemical perspective from the Reykjanes Ridge. *J. Petrol.* **43**, 1987-2012 (2002).
22. Shorttle, O. *et al*. Fe-XANES analyses of Reykjanes Ridge basalts: Implications for oceanic crust’s role in the solid Earth oxygen cycle. *Earth Planet. Sci. Lett.* **427**, 272-285 (2015).
23. Zellmer, G. F., Rubin, K. H., Gronvold, K., & Jurado-Chichay, Z. On the recent bimodal magmatic processes and their rates in the Torfajokull-Veidivotn area, Iceland. *Earth Planet. Sci. Lett.* **269**, 388-398 (2008).
24. Thomson, A., & Maclennan, J. The distribution of olivine compositions in Iceland basalts and picrites. *J. Petrol.* **54**, 745-768 (2013).
25. Melson, W. G., O’Hearn, T. O., & Jarosewich, E. A data brief on the Smithsonian Abyssal Volcanic Glass Data File. *Geochem. Geophys. Geosyst.* **3**, 1-11 (2002).
26. Bezos, A., & Humler, E. The Fe^3+^/ΣFe ratios of MORB glasses and their implications for mantle melting. *Geochim. Cosmochim. Acta* **69**, 711-725 (2005).
27. Christie, D. M., Carmichael, I. S. E., & Langmuir, C. H. Oxidation states of mid-ocean ridge basalt glasses. *Earth Planet. Sci. Lett.* **79**, 397-411 (1986).
28. Blichert-Toft, J. *et al*. Geochemical segmentation of the Mid-Atlantic Ridge north of Iceland and ridge-hot spot interaction in the North Atlantic. *Geochem. Geophys. Geosyst.* **6**; 10.1029/2004gc000788 (2005).
29. Kelley, K. A., Kingsley, R., & Schilling, J.-G. Composition of plume-influenced mid-ocean ridge lavas and glasses from the Mid-Atlantic Ridge, East Pacific Rise, Galapagos Spreading Center, and Gulf of Aden. *Geochem. Geophys. Geosyst.* **14**, 223-242 (2013).
30. Jenner, F. E., & O’Neill, H. S. C. Analysis of 60 elements in 616 ocean floor basaltic glasses. *Geochem. Geophys. Geosyst.* **13**, 1-11 (2012).
31. Reekie, C. *et al*. Sulfide resorption during crustal ascent and degassing of oceanic plateau basalts. *Nat. Commun.* **10**, 1-11 (2019).
